# Evidence of functional divergence in MSP7 paralogous proteins: a molecular-evolutionary and phylogenetic analysis

**DOI:** 10.1186/s12862-016-0830-x

**Published:** 2016-11-28

**Authors:** Diego Garzón-Ospina, Johanna Forero-Rodríguez, Manuel A. Patarroyo

**Affiliations:** 1 Molecular Biology and Immunology Department, Fundación Instituto de Inmunología de Colombia (FIDIC), Carrera 50#26-20, Bogotá, DC Colombia; 2 PhD Programme in Biomedical and Biological Sciences, Universidad del Rosario, Carrera 24#63C-69, Bogotá, DC Colombia; 3 School of Medicine and Health Sciences, Universidad del Rosario, Carrera 24#63C-69, Bogotá, DC Colombia

**Keywords:** *Plasmodium*, Multigene family, *msp7*, Episodic positive selection, Functional divergence, Relaxed selection, Intensified selection

## Abstract

**Background:**

The merozoite surface protein 7 (MSP7) is a *Plasmodium* protein which is involved in parasite invasion; the gene encoding it belongs to a multigene family. It has been proposed that MSP7 paralogues seem to be functionally redundant; however, recent experiments have suggested that they could have different roles.

**Results:**

The *msp7* multigene family has been described in newly available *Plasmodium* genomes; phylogenetic relationships were established in 12 species by using different molecular evolutionary approaches for assessing functional divergence amongst MSP7 members. Gene expansion and contraction rule *msp7* family evolution; however, some members could have had concerted evolution. Molecular evolutionary analysis showed that relaxed and/or intensified selection modulated *Plasmodium msp7* paralogous evolution. Furthermore, episodic diversifying selection and changes in evolutionary rates suggested that some paralogous proteins have diverged functionally.

**Conclusions:**

Even though *msp7* has mainly evolved in line with a birth-and-death evolutionary model, gene conversion has taken place between some paralogous genes allowing them to maintain their functional redundancy. On the other hand, the evolutionary rate of some MSP7 paralogs has become altered, as well as undergoing relaxed or intensified (positive) selection, suggesting functional divergence. This could mean that some MSP7s can form different parasite protein complexes and/or recognise different host receptors during parasite invasion. These results highlight the importance of this gene family in the *Plasmodium* genus.

**Electronic supplementary material:**

The online version of this article (doi:10.1186/s12862-016-0830-x) contains supplementary material, which is available to authorized users.

## Background

DNA duplication is an important source of novelty regarding evolution, providing the basis for new molecular activities [1, 2]. The genomes from the three kingdoms of life have been modulated by this mechanism, having multiple copies of genes [2]. Multigene families might evolve in line with a concerted or birth-and-death evolutionary model [3]; paralogous genes keep the same function in the former due to gene conversion whilst paralogous genes could lose or acquire a new function in a birth-and-death model. Since functional importance is highly correlated with evolutionary conservation [4, 5], molecular biologists have used evolutionary approaches to infer functional changes in paralogous genes/proteins using DNA/amino acid sequences [5–8].

Gene duplication seems to be recurrent in *Plasmodium* genus. These parasites are able to infect several vertebrates such as birds, rodents and primates. More than 200 species have been described to date. Clustering in different lineages occurs according to host (bird/reptile-parasite, rodent-parasite, monkey-parasite and hominid-parasite lineages) [9, 10]. Several genes produced by gene duplication are involved in host-cell invasion [11–14]. MSP7 is a merozoite surface protein encoded by a gene belonging to a multigene family located in chromosome 13 in hominid- and rodent-parasites but in chromosome 12 in monkey-parasites. This family has a different copy number amongst *Plasmodium* species [15, 16]. These genes are expressed simultaneously but they are independently regulated [17–20]. Functional assays have shown that *P. falciparum* MSP7 (MSP7I) is proteolytically processed; the resulting 22 kDa C-terminal region fragment is not covalently associated with MSP1 [7] and has cross-reactivity with other MSP7 proteins [17]. Furthermore, this fragment appears be involved in invasion by binding to red blood cells [21]. The C-terminal regions in *P. yoelii* from different MSP7s seem to be necessary to interact with the 83 kDa MSP1 fragment [17]. The MSP7 knockout reduces the normal growth rate of the mutant parasite in *P. berghei*; however, it becomes restored a few days later [22].

The *msp7* family appears to follow the birth-and-death evolutionary model; some gene copies have been maintained in the genome for a long time and others appear to be more recent [15]. This family has had a complex evolutionary history regarding *P. vivax*. The C-terminal region is involved in gene conversion [23]. Moreover, this region is highly conserved and under negative selection, suggesting functional/structural constraint [23–25]; by contrast, some *P. vivax* MSP7 proteins’ central regions have high genetic diversity, maintained by balancing selection, possibly as an immune evasion mechanism [23, 24].

Recent protein-protein interaction assays have shown that MSP7 proteins do not appear to bind to the same host receptor [26]; moreover, these proteins seem to be forming different protein complexes in the parasite [7, 27–30], maybe to perform different parasite-host interactions. Such results flout the functional redundancy hypothesis [15, 18]; functional divergence in MSP7 paralogs thus appears to be probable. This study has analysed data concerning *msp7* multigene family evolution, including 13 available *Plasmodium* genomes by evaluating their phylogenetic relationships and adopting different and new molecular evolutionary approaches for assessing functional divergence amongst MSP7 proteins.

## Methods

### Sequence data, alignments and phylogenetic tree reconstruction

Genome sequences from 11 *Plasmodium* species (and one subspecies, GenBank access number: *P. reichenowi,* GCA_000723685.1; *P. falciparum,* GCA_000002765.1; *P. vivax,* GCA_000002415.2; *P. cynomolgi,* GCA_000321355.1; *P. inui,* GCA_000524495.1; *P. knowlesi,* GCA_000006355.1; *P. coatneyi,* GCA_000725905.1*; P. chabaudi,* GCA_000003075.2; *P. vinckei,* GCA_000709005.1 and GCA_000524515.1; *P. yoelii,* GCA_000003085.2 and *P. berghei,* GCA_000005395.1) as well as the partial genome sequences from *P. gallinaceum* (Wellcome Trust Sanger Institute, http://www.sanger.ac.uk/resources/downloads/protozoa/plasmodium-gallinaceum.html) were analysed to obtain *msp7* multigene family genomic regions. The *msp7* gene copy number for these 13 genomes was established, as reported previously [15, 24].

All gene sequences found were used to deduce amino acid sequences by using Gene Runner software; these sequences were then screened to distinguish the MSP_7C domain using the Pfam server [31] (domain access number: PF12948). All amino acid sequences were then aligned using the MUSCLE algorithm [32] and manually edited by GeneDoc software [33]. The best amino acid substitution model was selected by Akaike’s information criterion using the ProtTest algorithm [34]; the JTT + G + F model was used to infer phylogenetic tress using maximum likelihood (ML) and Bayesian (BY) methods. RAxML was used for ML analysis [35] and topology reliability was evaluated by bootstrap, using 1000 replicates. A Metropolis-coupled Markov chain Monte Carlo (MCMC) algorithm was used for BY analysis [36] with MrBayes [37]. This analysis was run until reaching a standard deviation of split frequencies (ASDSF) value lower than 0.01; sump and sumt commands were used for tabulating posterior probabilities and building a consensus tree; in addition to ASDSF, the PSRF parameter was used for monitoring convergence. Both analyses were performed at CIPRES Science Gateway [38, 39]. A recent evolutionary multigene family model called DLRTS [40] was also performed; this method infers a gene tree by evolving down on a given species tree (with divergence times) by means of duplication, loss and transfer events according to a birth-death-like process [40, 41]. The species tree was inferred with a fragment of cytochrome C oxidase subunit 2 and divergence times were obtained from Pacheco et al. [42]. DLRTS was run using the MCMC algorithm for 10 million generations.

### Gene conversion amongst *msp7* members

It has been shown that some *msp7* family members (*msp7H* and *msp7I*) have evolved by gene conversion, thereby contributing to these members’ homogenisation [23]. *msp7* sequences were obtained from 6 *P. vivax* isolates (Salvador-I, Mauritania-I, India-VII, North Korean, Brazil-I and ctg isolate) [43, 44] and used to assess whether this pattern has also taken place in other *msp7* members. Betran’s method was used for gene conversion amongst paralogous genes [45] as well as the GENECONV algorithm [46]. DnaSP software was used for the former method [47] where only conversion tracts larger than 10 nucleotides were considered whilst RDP3 v3.4 software was used for GENECONV [48], considering just conversion tracts having *p* < 0.01. The same approach was followed for *P. falciparum msp7* genes, using the 3D7, FCR3, RO33, 7G8, K1, T9/102 and w2mef isolates.

### Identifying episodic diversifying selection on *msp7* tree branches

**Fig. 1:**
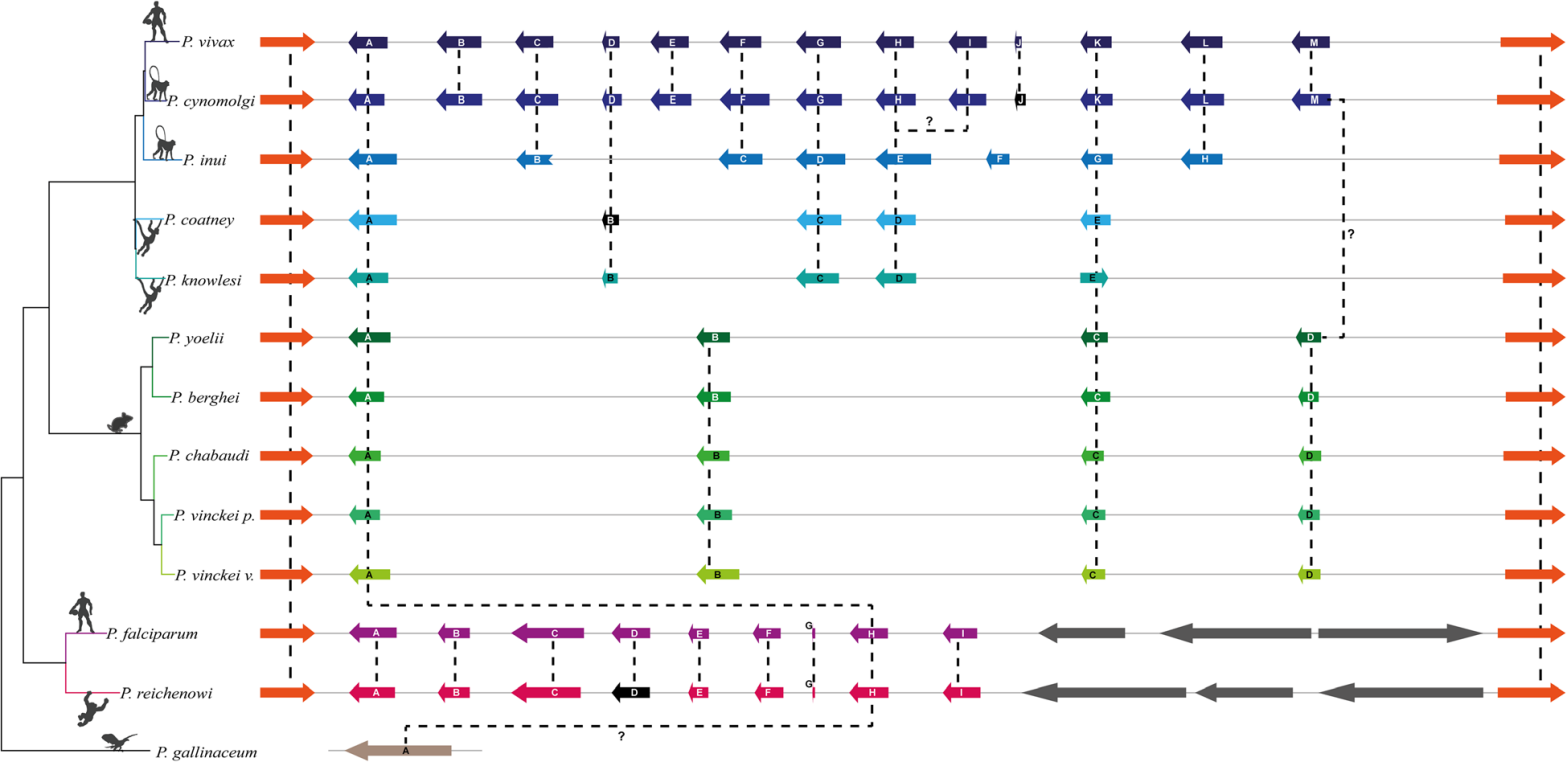
Schematic representation of the chromosomal *msp7* loci in 13 *Plasmodium* genomes. The genes flanking the *msp7* chromosome region in *Plasmodium* species are represented by *orange boxes*. The *coloured boxes* within flanking sequences represent *msp7* genes in each species whilst *black boxes* symbolise pseudogenes. The genes are given in alphabetical order from left to right. The dashed lines connect orthologous genes. All genes are represented to scale, but the distance between them is not representative. Question marks refer to what were not clearly orthologous relationships. By contrast with hominid-parasites, species from monkey-parasite and rodent-parasite lineages seem to have similar evolutionary histories regarding *msp7* expansion. The *grey boxes* are lineage-specific genes only found in hominid-parasite species, as they do not belong to the *msp7* family (the latter gene representations are not to scale)

**Fig. 2:**
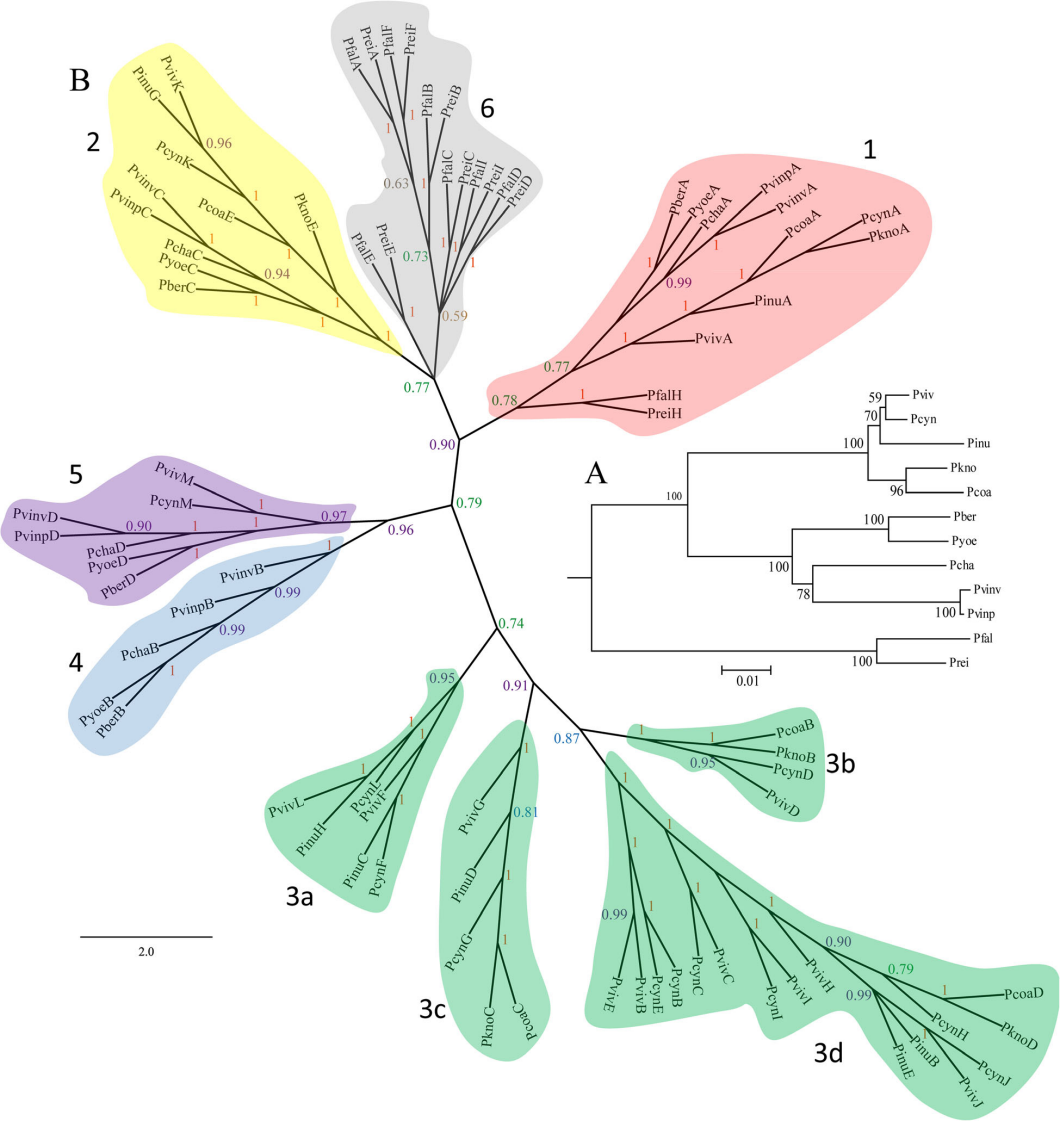
*msp7* gene family phylogeny inferred by the DLTRS evolutionary model. **a** Species tree used for generating the MSP7 tree. **b** MSP7 tree created by evolving down the species tree. *Numbers* represent different clades whilst numbers on branches are posterior probability values. Nine major clades were identified on the tree. Proteins were clustered in agreement with parasite phylogenetic relationships, clades 1 (*red*), 2 (*yellow*) and 5 (*purple*) being the most ancestral ones. The clades clustering genes from monkey-parasite lineage are depicted in green, proteins from rodent-parasite lineage in blue and hominid-parasite lineage in grey. The *P. inui* specie-specific duplicate was not considered in this analysis. Due to the family’s complex evolutionary history (which includes gene conversion, intragenic recombination, positive and/or balancing selection) the MCMC analysis did not converge and therefore the duplication/lost rates were not obtained even though a tree reconciliation similar to other topologies was inferred (BY and ML)

The random effects branch-site model (Branch-site REL) was used for assessing whether *msp7* multigene family lineages had been subject to episodic diversifying selection [49]. This method identifies branches where a percentage of sites have evolved under episodic diversifying selection. The MUSCLE algorithm was used for independently aligning each orthologous cluster’s amino acid sequences (Figs. 1 and 2); PAL2NAL software [50] was then used for inferring codon alignments from the aligned amino acid sequences. The best evolutionary models for DNA and protein alignments were inferred by using jModelTest [51] and ProtTest [34], respectively. ML phylogenetic trees were obtained for DNA and protein alignments for each orthologous cluster and used as phylogenetic framework to perform the Branch-site REL method using the HyPhy software package [52]; additionally, the Datamonkey web server [53] was also used to perform this method. The MEME method [54] from Datamonkey server was used to infer which sites were under episodic positive selection in each cluster.

### Evolutionary analysis for testing functional divergence

Functional redundancy was previously proposed for the MSP7 family by using sequences from seven species [15]. The sequence number in this research was increased and two different phylogeny-based approaches were used to assess functional divergence or redundancy between MSP7 members; one involved using DIVERGE v.3 software [55] to estimate type-I functional divergence [5] which is an indicator of functional changes between members of a multigene family [5, 6, 56–61]. This method is based on (site-specific) shifted evolutionary rates. It assesses whether there has been a significant change in evolution rate after duplication (or speciation) events by calculating the coefficient of divergence (θ_D_) and determining (e.g. by a Likelihood ratio test [5]) whether it is statistically significant for rejecting the null hypothesis (no functional divergence). The software then computes a posterior probability for detecting amino acids responsible for such divergence. Taking into account that new functions in paralogous proteins might emerge after gene duplication whenever selective strength is relaxed or whether positive selection is intensified, a second approach was used with the RELAX method [62]. This method allows partitioning a phylogeny into two subsets of branches to determine whether selective strength was relaxed or intensified in one of these subsets (test branch) relative to the other (reference branch). The Datamonkey web server was used for this analysis.

## Results

### *msp7* chromosomal locus genetic structure in *Plasmodium* spp

Whole genome sequences from 11 *Plasmodium* species, 1 subspecies and 1 partial draft genome sequence were screened for describing the *msp7* chromosomal locus. The *msp7* locus is circumscribed by PVX_082640 and PVX_082715 genes in *P. vivax* [15, 16, 24]; genes sharing high similarity to them were thus searched in the remaining species. Since MSP7 proteins appear be encoded by a single exon [63], the contigs enclosing flanking genes were analysed using ORFFinder and Gene Runner software to identify open reading frames (ORFs) encoding proteins larger than 200 amino acids. Seventy-nine ORFs (Additional file 1) in 13 genomes having the same transcription orientation were found (Fig. 1). These ORFs had 0.6 to 1.3 kilobases (kb) but *P. gallinaceum msp7* had a 3.1 kb length. Like previous studies [15, 16, 24], the copy number was different in *Plasmodium* spp. *P. vivax* and *P. cynomolgi* had the largest copy number (12 ORFs) whilst the lowest copy number was found in *P. gallinaceum* (just one gene). Shorter fragments (having more than 30% similarity with the identified ORFs) were also found in *P. vivax*, *P. cynomolgi*, *P. falciparum* and *P. reichenowi*. These ORFs (and small fragments) were named in alphabetical order regarding PVX_082640 and its homologous genes (Fig. 1 and Additional file 1).

Data regarding *P. inui*, *msp7B* (*pinumsp7B*) was incomplete due to gaps in the contig whilst *pcynmsp7J*, *pcynmsp7L*, *pcoamspB*, *preimsp7D* and *pvinvmsp7A* had premature stop codons. However, *pcynmsp7L* could encode a full MSP7 protein since it was shown to have intron donor/acceptor sites by GeneScan [64] screening, as previously shown [24]. Despite GeneScan not showing an intron/exon structure for *pvinvmsp7A*, it has putative donor/acceptor sites (Additional file 2). The Phobius algorithm was used for determining the presence of signal peptides within ORFs and Pfam for the MSP_7C domain; some genes did not have a signal peptide or the characteristic MSP_7C domain in the C-terminal region (Table 1).

**Table 1:** In-silico characterisation of putative MSP7 proteins

		*msp7* genes												
		A	B	C	D	E	F	G	H	I	J	K	L	M
*P. vivax*	SP	y	y	y	y	y	y	y	y	y	-	y	y	y
	MSP7_C	y	y	y	-	y	y	y	y	y	y	y	y	-
*P. cynomolgi*	SP	y	y	y	y	-	y	y	y	y	-	y	y	y
	MSP7_C	y	y	y	-	y	y	y	y	y	y	y	y	-
*P. inui*	SP	y	-	y	y	y	-	y	y					
	MSP7_C	y	y	y	y	y	-	y	y					
*P. knowlesi*	SP	y	y	y	y	y								
	MSP7_C	y	-	y	y	y								
*P. coatneyi*	SP	y	-	y	y	y								
	MSP7_C	y	-	y	y	y								
*P. chabaudi*	SP	y	y	y	y									
	MSP7_C	y	y	y	-									
*P. vinckei v.*	SP	y	y	y	y									
	MSP7_C	y	y	y	-									
*P. vinckei p.*	SP	y	y	y	y									
	MSP7_C	y	y	y	-									
*P. berghei*	SP	y	y	y	y									
	MSP7_C	y	y	y	-									
*P. yoelii*	SP	y	y	y	y									
	MSP7_C	y	y	y	-									
*P. falciparum*	SP	y	y	-	y	y	y	y						
	MSP7_C	y	y	y	y	y	y	y	y	y				
*P. reichenowi*	SP	y	-	y	y	y	y	y						
	MSP7_C	y	y	y	y	y	y	y	y	y				
*P. gallinaceum*	SP	y												
	MSP7_C	y												

Eighty-three sequences between flanking genes were screening for identifying a signal peptide and the characteristic MSP_7C domain (Pfam access number: PF12948). y: proteins having a signal peptide according to the Phobius algorithm or a MSP_7C domain in a Pfam search. -: proteins appeared not to have a signal peptide or MSP_7C domain

### The *Plasmodium msp7* family’s phylogenetic relationships

Phylogenetic relationships for this family were identified as previously described [15, 24, 65, 66]. A multiple alignment was performed for deducing 80 *msp7* genes’ amino acid sequences (excluding the shorter gene fragments and *P. gallinaceum* gene). A phylogenetic tree was then inferred by using ML and BY methods with the JTT + G + F model; both topologies gave similar branch patterns, displaying 12 major clades (Additional file 3), with clade 1 clustering sequences from 12 *Plasmodium* species considered in this study, clade 2 another 10 of them and clade 5 clustering the last gene in the chromosomal region from rodent-parasites, *P. vivax* and *P. cynomolgi*. The remaining clades put together sequences according to *Plasmodium* lineages (i.e. MSP7 sequences from the monkey-parasite lineage were in clades 3, clade 4 clustered the sequences from rodent-parasite lineage and clades 6 clustered sequences from the hominid-parasite lineage). Clades 1, 2, 3a, 3b, 4 and 6b only contained orthologous proteins whilst clades 3c and 3d had orthologous and paralogous proteins from monkey-parasite lineage, the clade 6a clustered sequences from hominid-parasite lineage whilst the sequence in clade 7 appeared to be exclusive to *P. inui*. In addition to previous studies [15], the DLTRS model was implemented which reconciles the gene tree to the species tree [40, 41]. The fraction of sampled values discarded as burn-in during analysis was 0.25 but the MCMC chain did not converge after more than 10 million generations, therefore, gene duplication, loss or transfer rates were not obtained. However, the reconciliation of the *msp7* gene tree to the *Plasmodium* species tree was obtained. The tree inferred by DLTRS had 9 major clades (Fig. 2), thereby agreeing with the ML and BY clades (Fig. 2 and Additional file 3). In all phylogenies (Fig. 2 and Additional file 3) the sequences without the MSP_7C domain (PvivMSP7D, PcynMSP7D, PknoMSP7B and PcoaMSP7B) appeared to be phylogenetically related to sequences having an MSP_7C domain. However, this relationship was only supported by posterior probabilities but not by bootstrapping (Fig. 2 and Additional file 3). Furthermore, these sequences, like others not containing an MSP_7C domain, had noticeable similarity (>48%) with MSP7 members at the N-terminal end (Additional file 4).

Since PgalMSP7A is larger than other MSP7s, we did not take it into account for the aforementioned analysis; then, only the MSP_7C domain was used for inferring their phylogenetic relationships to determine whether PgalMSP7A was orthologous to MSP7A/H. The branch pattern from this topology (Additional file 5) was similar to the phylogeny obtained by using all sequences (Fig. 2, Additional files 3 and 5). PgalMSP7A clustered with MSP7A; however, posterior probability and bootstrapping were low. PgalMSP7A did not cluster with any other MSP7 in DLTRS tree; instead it appeared as an outgroup.

### Gene conversion amongst *msp7* genes

**Fig. 3:**
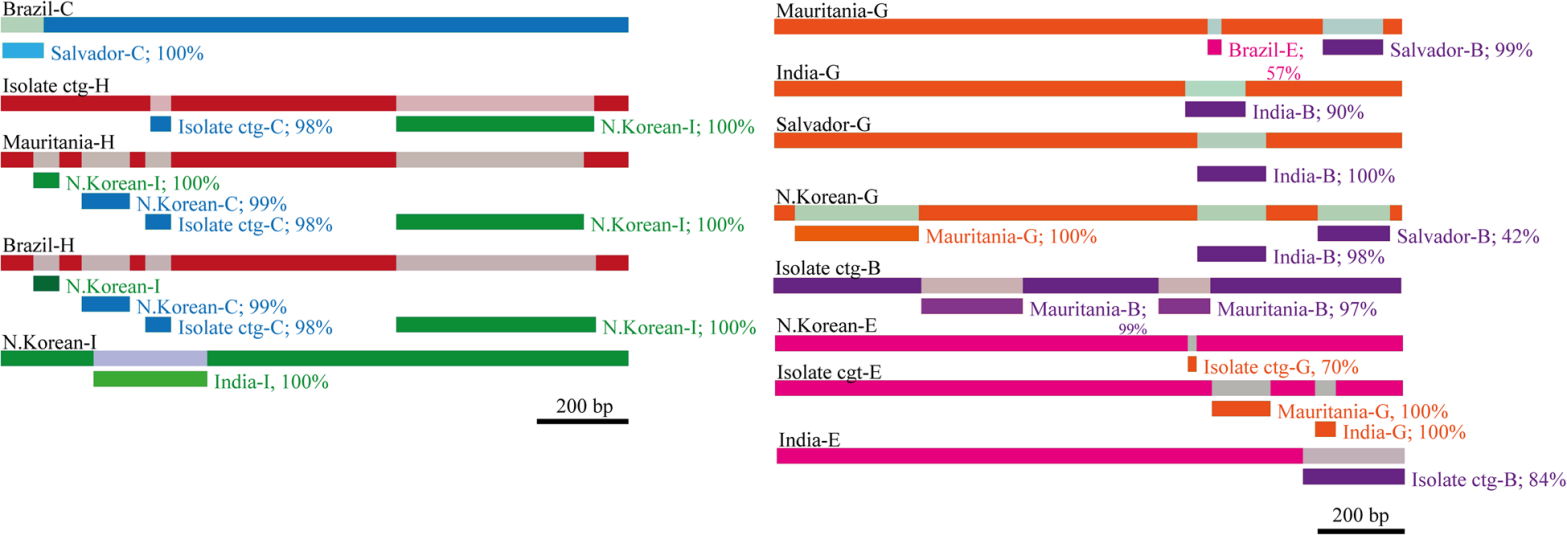
Schematic representations of gene conversion tracks identified by the GENECONV method. Each gene is represented by a colour bar (*pvivmsp7C* (*blue*), *pvivmsp7H* (*red*), *pvivmsp7I* (*green*), *pvivmsp7B* (*purple*), *pvivmsp7E* (*fuchsia*), and *pvivmsp7G* (*orange*)); a different *coloured rectangle* is a graphical representation of sequence fragments potentially originating from gene conversion. Conversion tracks were mainly located in the 3′-ends. The % value refers to the similarity value for the sequence region involved in gene conversion (or intragenic recombination)

An atypical pattern in the clades clustering MSP7H/7I and MSP7B/7E/7G was observed in the phylogenies inferred above (Fig. 2, Additional files 3 and 5). Contrary to what would have been expected, PvivMSP7B was clustered with PvivMSP7E and PvivMSP7G and not with their respective orthologues; likewise, PvivMSP7H and PvivMSP7I seemed to share a common origin. It has previously been reported that gene conversion takes place between PvivMSP7H and 7I [23]. We obtained the *pvivmsp7*s sequences from 6 *P. vivax* isolates to assess whether gene conversion takes place in PvivMSP7B, 7E and 7G. Alignments for *pvivmsp7C*, *7H* and *7I* and another for *pvivmsp7B*, *7E* and *7G* were performed; Betran’s method and the GENECONV algorithm were then used, displaying recombination tracks between isolates but also between paralogous genes. The Betran algorithm found 2 conversion tracks between *pvivmsp7C* and *7I*, whilst there were 3 conversion tracks between *pvivmsp7B* and *7E* and another two between *pvivmsp7E* and *7G*. Figure 3 shows the conversion tracks found by GENECONV. The same approach was followed for *P. falciparum* by using different reference isolates. By contrast with *P. vivax, pfmsp7* members did not seem to be affected by gene conversion since no conversion tracks were found amongst them (data not shown).

### Episodic positive selection on *msp7* branches

**Fig. 4:**
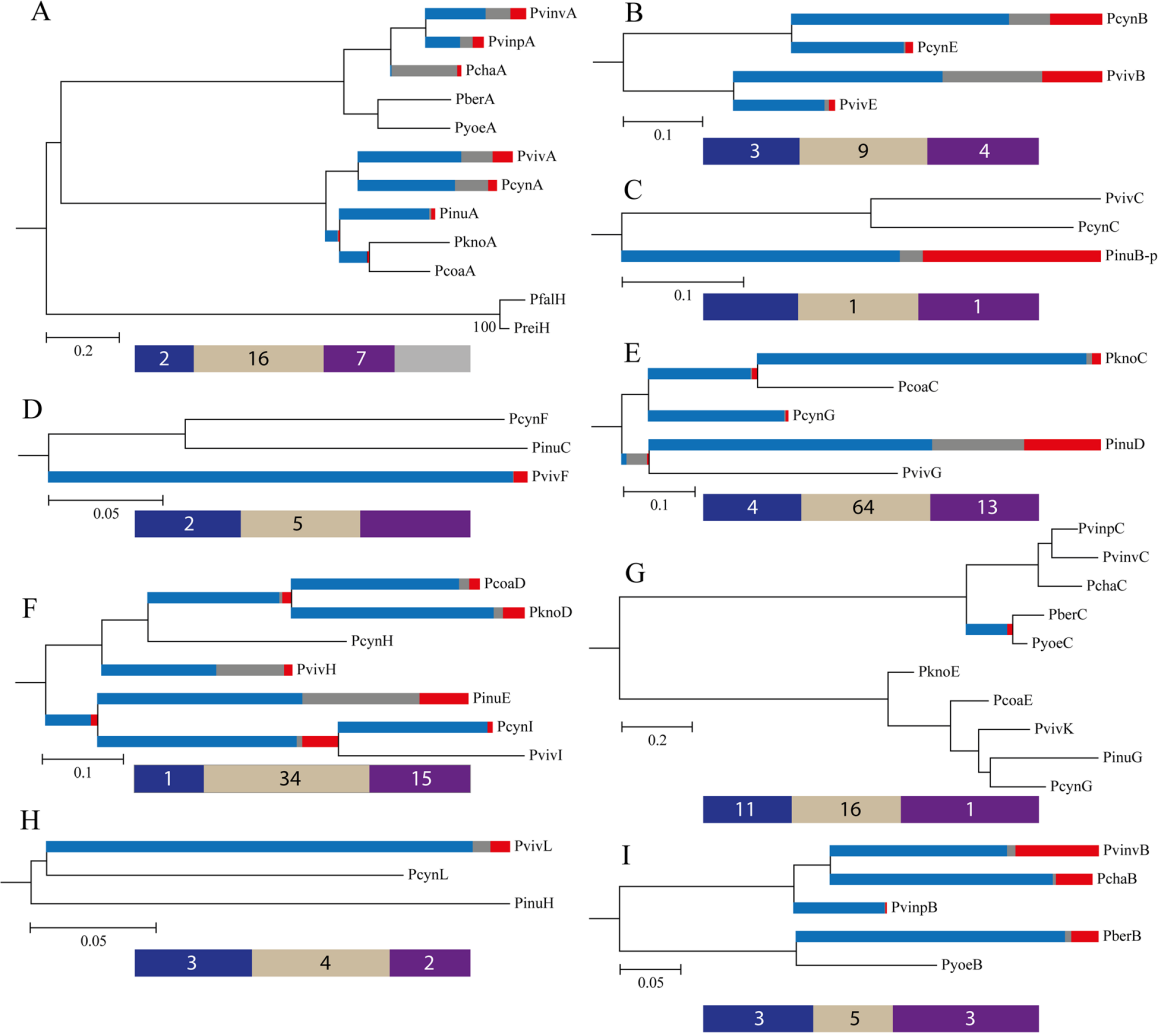
Phylogenies analysed for episodic selection. Each orthologous cluster was analysed by the Branch-site REL method. The shade of each colour on branches indicates strength of selection (*red* shows ω >13, *blue* ω ≤1 and *grey* ω = 1). The size of each colour represents the percentage of sites in the corresponding class found by Branch-site REL. Branches have been classified as undergoing episodic diversifying selection by the *p*-value corrected for multiple testing using the Holm-Bonferroni method at *p* < 0.05. **a**. clade 1; **b**. *pviv/pcynmsp7B* and *7E*; **c**. *pviv/pcynmsp7C* and *pinumsp7B*; **d**. *pviv/pcynmsp7F* and *pinumsp7C*; **e**. *pviv/pcynmsp7G*, *pkno/pcoamsp7C* and *pinumsp7D*; **f**. *pviv/pcynmsp7H/7I*, *pkno/pcoamsp7D* and *pinumsp7E*; **g**. clade 2; **h**. *pviv/pcynmsp7L* and *pinumsp7H* and **i**. clade 4. At the bottom of each phylogeny there is a scale representation of *msp7s*. The *blue boxes* represent the encoded N-terminal region, the *light brown* ones symbolise the central region and the *purple boxes* the MSP_7C domain. Numbers within boxes represent the number of codons under positive selection inferred by MEME, SLAC, FEL, REL and FUBAR methods using the Datamonkey web server

Previous studies have shown ancestral positive selection regarding the *msp1* gene in the monkey-parasite lineage [67, 68]. Such episodic positive selection could have occurred in order to adapt to newly-appeared macaque species [67]. Since MSP1 and MSP7 form a protein complex [7, 18, 29] involved in parasite invasion, both proteins should have similar selective pressures. Since this has been assessed just for *msp7E* and *7L* [24], the Branch-site REL method was used here for assessing whether other lineages are subject to episodic diversifying selection in *msp7* evolutionary history; evidence was found of strong episodic diversifying selection in a few internal branches and in several external branches (Fig. 4 and Additional file 6). Regarding rodent-parasites, the lineages leading to MSP7A and 7B in *P. vinckei vinckei*; *P. vinckei petteri*, *P. chabaudi* (Fig. 4a) and *P. berghei* (Fig. 4i) were under selection. Just one internal branch (the *P. berghei*/*P. yoelii* MSP7C ancestor, Fig. 4g) displayed episodic selection. Concerning monkey-parasites, a percentage of sites regarding the lineages leading to MSP7A in *P. vivax*, *P. cynomolgi* and *P. inui* as well as the *P. inui*/*P. knowlesi/P. coatneyi* and *P. knowlesi*/*P. coatneyi* lineage ancestors (Fig. 4a) were under very strong episodic positive selection (ω > 33). Likewise, the lineages that gave rise to *P. cynomolgi* MSP7B, 7E, *P. vivax* MSP7B, 7E (Fig. 4b); *P. inui* 7B (Fig. 4c); *P. vivax* 7F (Fig. 4d); *P. knowlesi* 7C, *P. cynomolgi* 7G, *P. inui* 7D (Fig. 4e); *P. coatneyi* 7D, *P. knowlesi* 7D, *P. vivax* 7H, *P. inui* 7E, *P. cynomolgi* 7I (Fig. 4f) and *P. vivax* 7L (Fig. 4h) also were under positive selection. Very strong episodic positive selection was observed in the *P. knowlesi*/*P. coatneyi* 7C, *P. inui* 7D/*P. vivax* 7G, *P. coatneyi*/*P. knowlesi* 7D, *P. inui* 7E/*P. cynomolgi 7I*/*P. vivax* 7I and *P. cynomolgi*/*P. vivax* MSP7I ancestral branches (Fig. 4 and Additional file 6).

### Evolutionary analysis for testing functional divergence

Gu’s type-I functional divergence and RELAX methods were used to identify functional divergence amongst MSP7 proteins. Pairwise comparisons between paralogous proteins (e.g. from different clades) as well as between orthologues (e.g. proteins from different parasite lineage but within the same clade), showed high coefficient of divergence (θ_D_) values (Table 2); between 10 and 20% of the sites had a significant change in their evolutionary rate (Additional file 7). Likewise, we found either a relaxed selection or an intensified selection amongst MSP7 paralogues (Table 2 and Additional file 8).

**Table 2:** *In silico* assessment of functional divergence between paralogous and orthologous MSP7 proteins

#	Cluster A	Compared to	Cluster B	θ_D_	LRT_θD_	RELAX (*p*-value)
1	Clade 1 Primate-parasites		Clade 1 Rodent-parasites	0.54	4.74^a^	NP
2	Clade 1 Primate-parasites		Clade 3d Primate-parasites (B/E)	−0.21	0.00	Intensification (0.0038)
3	Clade 1 Rodent-parasites		Clade 3d Primate-parasites (B/E)	0.78	15.19^a^	NP
4	Clade 1 Primate-parasites		Clade 3d Primate-parasites (C/H/E/D/I)	0.34	4.25^a^	Intensification (0.00006)
5	Clade 1 Primate-parasites		Clade 3c Primate-parasites (G/C/D)	0.90	14.46^a^	Relaxation (1)
6	Clade 1 Rodent-parasites		Clade 3c Primate-parasites (G/C/D)	0.54	8.89^a^	NP
7	Clade 1 Rodent-parasites		Clade 3d Primate-parasites (C/H/E/D/I)	0.37	13.01^a^	NP
8	Clade 1 Primate-parasites		Clade 2 Primate-parasites	0.45	3.79	Relaxation (0,2)
9	Clade 1 Primate-parasites		Clade 2 Rodent-parasites	0.20	0.76	NP
10	Clade 1 Rodent-parasites		Clade 2 Primate-parasites	0.74	13.21^a^	NP
11	Clade 1 Rodent-parasites		Clade 2 Rodent-parasites	0.93	24.39^a^	Intensification (9.1e-7)
12	Clade 2 Primate-parasites		Clade 2 Rodent-parasites	0.87	12.60^a^	NP
13	Clade 1 Primate-parasites		Clade 4 Rodent-parasites	0.15	0.14	NP
14	Clade 1 Rodent-parasites		Clade 4 Rodent-parasites	0.69	22.20^a^	Relaxation (0.4)
15	Clade 3d Primate-parasites (B/E)		Clade 3d Primate-parasites (C/H/E/D/I)	0.03	0.14	Intensification (0.04)
16	Clade 3d Primate-parasites (B/E)		Clade 2 Primate-parasites	0.72	18.91^a^	Intensification
17	Clade 3d Primate-parasites (B/E)		Clade 2 Rodent-parasites	0.31	4.99^a^	NP
18	Clade 3d Primate-parasites (B/E)		Clade 4 Rodent-parasites	0.35	7.76^a^	NP
19	Clade 3d Primate-parasites (B/E)		Clade 3c Primate-parasites (G/C/D)	0.37	3.91^a^	Intensification (2.8e-7)
20	Clade 2 Primate-parasites		Clade 3d Primate-parasites (C/H/E/D/I)	0.93	37.86^a^	Intensification (0.0002)
21	Clade 3d Primate-parasites (C/H/E/D/I)		Clade 2 Rodent-parasites	0.81	20.90^a^	NP
22	Clade 3d Primate-parasites (C/H/E/D/I)		Clade 4 Rodent-parasites	0.72	15.04^a^	NP
23	Clade 2 Primate-parasites		Clade 4 Rodent-parasites	1.0	27.64^a^	NP
24	Clade 3c Primate-parasites (G/C/D)		Clade 3d Primate-parasites (C/H/E/D/I)	0.45	9.55^a^	Relaxation (0.000008)
25	Clade 2 Primate-parasites		Clade 3c Primate-parasites (G/C/D)	1.0	23.68^a^	Relaxation (0.5)
26	Clade 3c Primate-parasites (G/C/D)		Clade 2 Rodent-parasites	0.46	3.04	NP
27	Clade 3c Primate-parasites (G/C/D)		Clade 4 Rodent-parasites	0.44	3.94^a^	NP
28	Clade 4 Rodent-parasites		Clade 2 Rodent-parasites	0.57	8.56^a^	Intensification (0.03)

The coefficients of divergence (θ_D_) and their LRT values from pairwise cluster comparisons in the *msp7* multigene family. LRT_θD_ is the (log) score for the likelihood ratio test against the null hypothesis (θ_D_ = 0) [5]. It is the output of DIVERGE and it follows a chi-square distribution with one degree of freedom; thus, values greater than or equal to 3.84 (^a^) indicate functional divergence between pairwise clusters. Selection intensity (relaxation or intensification) found by the RELAX method is shown for paralogous pairwise comparisons (see Additional file 8). Comparisons 2, 4 and 15 revealed fewer positive selected sites on test branches than on reference branches as well as an intensification of negative selected sites and non-significant θ_D_. Comparisons 11, 19, 20 and 28 revealed an increased proportion of positive selected sites on the test branches, having an intensification of this kind of selection, while the proportion of negative selected sites stayed the same or decreased. The θ_D_ values were statistically significant. Comparisons 5 and 24 gave a statistically significant θ_D_ and relaxed selection (on test branches). NP: analysis was not performed because proteins came from different species

## Discussion

It has been suggested that DNA duplication is the main source of evolutionary innovation [1, 2] since duplicate DNA fragments might evolve to new functions. However, acquiring new functions (neofunctionalisation) is not always the duplicate’s outcome [69]. There are other fates for paralogous fragments such as non-functionalisation (or pseudogenisation), subfunctionalisation [69] or even functional redundancy. Encoded-protein gene duplication in parasitic organisms involved in host recognition could provide an advantage, regardless of whether such duplicates increase host recognition ability. *Plasmodium* genomes have a lot of genes as multigene families [11–14, 44], some of them are functionally equivalent whilst others have functionally diverged (they recognise different host receptors) [70–73].

The *msp7* family has been previously described in eight species [15, 16, 24], displaying different expansion. The present study has analysed 4 new species and completed analysis for *P. reichenowi*. An unequal copy gene number was found in *Plasmodium* species, suggesting lineage or specie-specific duplications or deletions. We also found genes which have become pseudogenes, thereby confirming the birth-and-death model of evolution for this family (Fig. 1). Then again, phylogenetic analysis was used for establishing phylogenetic relationships amongst *msp7* paralogues. According to the phylogenetic trees (Fig. 2 and Additional file 3), MSP7A/H (clade 1) was the most ancestral gene, followed by clade 2 which is shared by monkey- and rodent-parasites. Clade 5 was also an old clade since it is shared amongst monkey- and rodent-parasites; however, not all monkey-parasites had this copy and it thus became lost in *P. knowlesi*, *P. coatneyi* and *P. inui*. Moreover, these proteins in monkey- and rodent-parasites did not have the MSP_7C domain. The remaining clades were clustered in agreement with *Plasmodium* species’ relationships (e.g. *P. vivax* genes clustered with *P. cynomolgi* genes) and they were syntenic, suggesting they are orthologues. These expansions reproduced the genus phylogenetic relationships. Species belonging to monkey-parasite lineage had the highest copy number. *P. vivax* and *P. cynomolgi* (sister taxa) shared the whole *msp7* repertory. *P. inui* is the phylogenetically closest species to the aforementioned ones, having 7 orthologues followed by *P. knowlesi* and *P. coatneyi* having 5 orthologues. The latter species are sister taxa sharing a common ancestor as well as the whole *msp7* repertory. The monkey-parasite lineage is a sister taxon to rodent-parasite lineage. At least two orthologous genes were found within these two lineages whilst only one gene was shared between these and the hominid-parasite lineage (Fig. 1). The most ancient *Plasmodium* lineage is the bird/reptile-parasite. We analysed *P. gallinaceum* and found just one large *msp7* gene. According to the MSP7 C-terminal phylogenetic tree (Additional file 5), it is still unclear whether this large gene is orthologous to the most ancestral gene (*msp7A*/*H*). As we did not find any more *msp7* genes in the *P. gallinaceum* partial genome, gene expansion should have taken place after mammal-parasite radiation 40 million year ago [42].

We found 83 sequences between bordering genes in the 13 genomes (Fig. 1 and Additional file 1); however, 11 protein sequences did not have the MSP_7C domain at the C-terminal end, though some of them did cluster with MSP7 proteins (those containing the MSP_7C domain). Proteins lacking such domain had high similarity with MSP7s at the N-terminal end (Additional file 4). This suggested that proteins lacking an MSP_7C domain (MSP7-like) are incomplete duplicates or have lost the domain throughout *Plasmodium* evolutionary history.

On the other hand, groups clustering paralogous proteins (3d clade) displayed an unusual pattern (Fig. 2 and Additional file 3). Proteins such as Pviv/PcynMSP7E and Pviv/PcynMSP7B seemed to be more similar within species than between species. A similar branch pattern was observed in the MSP_7C phylogenetic tree (Additional file 5), where PvivMSP7B was more similar to PvivMSP7E than PcynMSP7B. Likewise, PvivMSP7H was more similar to PvivMSP7I whilst PcynMSP7H clustered together with PcynMSP7I. A previous study has shown that gene conversion takes place in *pvivmsp7H* and *7I* genes [23]; such branching pattern is therefore a consequence of gene conversion. We also observed gene conversion tracks amongst *P. vivax* reference isolates in the aforementioned genes, suggesting that this mechanism also occurs in *pvivmsp7B*, *7E* and *7G*; however, such genes are not near each other (Fig. 1) and there was no complete gene homogenisation. Therefore, it is not clear whether this mechanism is taking place at present or they are ancient gene conversion events.

Parasite invasion involves several protein-protein interactions between parasite and host. MSP1 is the protein mediating initial interaction, this protein and MSP7 form a complex involved in parasite-host interaction [7, 18, 21, 29]. MSP1 has shown an episodic positive selection signal throughout its evolutionary history [67, 68]; such positive selection is shown at ancestral branches and is likely the result of adapting to newly-appeared macaque species 3.7–5.1 million years ago [67] or during human switching [74]. Since MSP7 is in a complex with MSP1 [7, 18, 29], the former should have similar selective pressures and therefore similar behaviour. We have found several lineages under strong diversifying selection (ω > 10 [49], Additional file 6). As in MSP1 [67, 68], few internal branches were under episodic selection (Fig. 4a, e, f and g); this pattern could be the outcome of adaptation to new hosts during *Plasmodium* sympatric speciation, as has been suggested for other antigens [67, 68]. On the other hand, several external lineages (branches) were under selection. This could be the outcome of changes in evolutionary rates throughout *msp7* paralogous evolution which have been favoured by selection since they may have promoted adaptation to a new host or acquiring new molecular activities.

Previous work did not recognise codons under positive selection [15]; however, here we identified codons under selection by using improved and/or newly developed methods (Fig. 4). The greatest amount of codons under positive selection was located in central *msp7* regions and a few at 5′ or 3′-ends. Population genetics studies have shown the central region to be the most polymorphic and it seems to be involved in immune evasion [23, 24]. The positive selected sites found amongst species in central regions could thus be the consequence of host adaptation to avoid host immune responses. On the other hand, positive selected sites at the encoding C-terminal region could be the outcome of coevolution between host receptor and parasite MSP7 ligands and also the result of the acquisition of new roles (Additional file 8).

Despite functional redundancy having been suggested for the MSP7 family [15, 18], some groups have shown that MSP7 proteins appear not to bind to the same host receptor [26]. Likewise, some PvivMSP7 proteins seem to be forming different protein complexes in the parasite [7, 27–30] which might allow different parasite-host interactions. We could not find evidence of functional divergence in MSP7 paralogues when comparing different clades (e.g. clade 1 against clade 2) in a previous in silico study [15]. This could have been because orthologous proteins might use different regions to interact with a host or with their own parasite proteins. The *P. falciparum* (hominid-parasite lineage) MSP1 region involved in host recognition is the 19 kDa fragment [75]; nevertheless, the 33 kDa fragment in *P. vivax* (monkey-parasite lineage) facilitates parasite-host interaction [76]. Therefore, even though they are orthologous, both proteins have differences in their evolutionary rates within functional regions [77]. Whether this behaviour also took place in MSP7, DIVERGE gave false negatives regarding functional divergence. We thus analysed MSP7 proteins from other *Plasmodium* species to assess functional divergence amongst MSP7 clades (paralogous) but also within clades (orthologous). Unlike a previous study [15], just the MSP7 C-terminal region was analysed here, taking into account that this region has the domain (MSP_7C) defining members of this family, it is the only MSP7 region in the protein complex [7], in *P. vivax* MSP7s C-terminal regions are highly conserved and under negative selection whilst other regions are highly polymorphic [23–25] and most regions binding to red blood cells are in the MSP7 C-terminal region [21]. We have found changes in evolutionary rates amongst paralogous proteins in this region. The coefficients of divergence (θ_D_) were statistically significantly larger than 0 between some clades (Table 2 and Additional file 7), suggesting that some MSP7s have diverged functionally. Such divergence could mean that different MSP7 proteins could form different parasite protein complexes or that MPS7s could interact with different host receptors. We also found changes in evolutionary rates within clades (between orthologues) leading to large θ_D_ values (e.g. between monkey-parasite MSP7A and rodent-parasite MSP7A). This could have been due to functional divergence or different protein regions carrying out the function (as previously shown for MSP1 [75, 76]).

It has been demonstrated that duplicated genes experience a brief period of relaxed selection early in their history [69]. This relaxation could have led to pseudogenisation or, rarely, evolving to new functions [69]. Moreover, positive selection in duplicates could also lead to them acquiring new molecular activities [62]; relaxed selection or intensification of positive selection must thus be identified in MSP7s having functional divergence. Our DIVERGE results were consistent with RELAX results (Table 2). Proteins showing functional divergence also displayed functional constraint relaxation or intensification of positive selection. However, some clades having high θ_D_ did not show relaxation or intensification. This could have been due to episodic positive selection. This kind of selection acts very quickly and involves a switch from negative selection to positive selection and back to negative selection [62]. Episodic selection was found in all *msp7* paralogous clusters by two different approaches (Fig. 4, Additional files 6 and 7); consequently, episodic positive selection allowing functional divergence could not have been detected by RELAX.

On the other hand, some paralogous proteins had no statistical θ_D_ values; they also displayed intensification of negative selection, thereby suggesting that they are functionally equivalent. Furthermore, functional redundancy in MSP7H, 7I and/or 7B and 7E could be favoured by gene conversion; some MSP7 members could therefore evolve by “partial gene conversion” affecting some but not all MSP7 paralogous proteins.

## Conclusion

We have described the *msp7* family in different *Plasmodium* species, using different phylogenetic and molecular evolution analyses. Although *msp7* evolved mainly in line with a birth-and-death evolutionary model, some members have evolved in a concerted way. Gene conversion has taken place between some paralogous genes allowing gene sequence homogenisation, these paralogous genes consequently keeping the same function. However, some gene conversion tracks could be ancient and thus the homogenisation has been lost. In addition, some paralogous proteins did not show changes in their evolutionary rates; thus, MSP7A, 7B, 7C, 7E, 7H and 7I in monkey-parasites seem to be functionally equivalent copies. Other MSP7 members showed alteration in their evolutionary rate as well as relaxed or intensified (positive) selection; functional divergence may thus have occurred in them. Such functional divergence could enable MSP7F, 7G, 7K and 7L (from monkey-parasites) and MSP7A, 7B and 7C (from rodent-parasites) to form different parasite protein complexes and/or recognise different host receptors during invasion. In fact, protein-protein assays have shown that PvivMSP7A interacts with PvivTRAg56.2 [30] whilst PvivMSP7L has been found forming a complex with a member of the MSP3 family [27]. Moreover, PvivMSP7G (but not 7C or 7L) is able to bind to human P-selectin whilst PberMSP7C binds better to mouse P-selectin than PberMSP7A [26]. The results described here highlight this family’s importance in the *Plasmodium* genus. Further functional assays should be performed based on these results to gain a deeper understanding of the biology of *Plasmodium* invasion.

## Additional files

Additional file 1: Eighty-tree sequences from MSP7 family found in 13 *Plasmodium* genomes. The PlasmoDB accession numbers for *msp7* genes from eight species are shown. (TXT 88 kb)
Additional file 2: Putative donor/acceptor sites in *P. vinckei vinckei msp7A*. (PDF 42 kb)
Additional file 3:
*msp7* gene family phylogeny inferred by the maximum likelihood method. Numbers represent different clades (based on Fig. 2 numbers from main text) whilst numbers on branches are bootstrap and posterior probability values. Thirteen major clades were identified on the tree; however, clades 1 and 6 were not clearly supported by bootstrap values. Proteins were clustered in agreement with parasite phylogenetic relationships, clades 1 (red) and 2 (yellow) being the most ancestral ones; clade 5 could also be an old cluster. The clades clustering genes from monkey-parasite lineage are depicted in green, proteins from rodent-parasite lineage in blue and hominid-parasite lineage in grey. The protein cluster in brown is a *P. inui* specie-specific duplicate. Both ML and BY trees showed similar topologies, but only the ML tree is shown. Some internal branches were inconsistent between BY and ML trees. Since *msp7* family has a complex evolutionary history (having gene conversion, intragenic recombination and/or natural selection) the tree inference is affected. However, external branches were consistent in both topologies as well as in Fig 2’s tree. In BY analysis, the settings for prior and likelihood were: prset aamodel = fixed (JONES); prset statefreqpr = fixed (empirical); lset rates = gamma (JTT + G + F). More than 11 million generations were required for reaching an ASDSF value lower than 0.01. The PSRF parameter was also used for monitoring convergence (PSRF: 1.000). (TIF 3836 kb)
Additional file 4: Similarity values in the N-terminal region between MSP7 proteins and sequences lacking an MSP_7C domain. (PDF 24 kb)
Additional file 5: Phylogenetic tree inferred with the C-terminal (MSP_7C) domain. Mammal-parasite MSP7 sequences containing the MSP_7C domain and the PgalMSP7 C-terminal domain were aligned and phylogenetic trees were then inferred. PgalMSP7 clustered with the most ancestral MSP7 protein (clade 1, see Fig. 2 in the main text) though this group was not supported by bootstrap and/or posterior probability. The remaining sequences clustered according to host-parasite lineages. Numbers on branches show bootstrap and posterior probabilities values. Clades shown in red and yellow were the most ancestral ones. The clades clustering genes from monkey-parasite lineage are depicted in green, proteins from rodent-parasite lineage in blue and hominid-parasite lineage in grey. Both ML and BY trees showed similar topologies but only the ML tree is shown. Numbers outside the clades represent the number of clades in Fig. 2 from the main text. (TIF 3038 kb)
Additional file 6: Episodic positive selection on MSP7 branches. ω + values reflect the maximum likelihood estimate rate of positive selection. *p*-value obtained after Holm-Bonferroni multiple testing correction. The Branch-site REL method was performed by HyPhy software using both amino acid and DNA phylogenies. The Datamonkey web server was also used for calculating this method. The number of sites under episodic positive selection was identified by MEME using Datamonkey. The letters in the first panel correspond to the letters in Fig. 4 from the main text. (PDF 292 kb)
Additional file 7: Putative sites involved in functional divergence. DIVERGE computed a posterior probability for detecting putative amino acids responsible for functional divergence in pairwise comparisons having statistical significant θ_D_ values. Such putative sites were highlighted in green and sites in the C-terminal region (MSP_7C domain) under positive selection found by the MEME method were tagged with a red plus symbol (+). Since functional divergence could involve relaxation of functional constraint or could be due to positive selection, a perfect correlation between putative amino acids responsible for divergence and positive selection would not have been expected. Positive selection might be involved in the acquisition of a new role but could also be the outcome of adaptation to a new host during *Plasmodium*’s evolutionary history. Positive selected sites were inferred using the clade (e.g. sequences within clade 1) but not using the sequences from the comparison (e.g. Clade 1 Primate-parasites vs Clade 2 Primate-parasites). (PDF 478 kb)
Additional file 8: Selection intensity (functional constraint relaxation or selection intensification) throughout *msp7* paralogous genes using the partitioned descriptive model. Three ω parameters (positive, negative and neutral) and the relative proportion of sites are plotted for test (blue) and reference (red) branches. The grey vertical dashed line at ω = 1 represents neutral evolution. The numbers in parenthesis indicate the comparison number in Table 2 from the main text. Comparisons 2, 4 and 15 show that the percentage of positive selected sites decreased on test branches; they also showed an intensification of negative selected sites (on test branches), having non-significant θ_D_. These results suggested functional redundancy. Comparisons 11, 19, 20 and 28 displayed an increased percentage of positive selected sites having an intensification of this kind of selection on the test branches whilst the percentage of negative selected sites remained equivalent or decreased. The θ_D_ values in these comparisons were statistically significant, suggesting functional divergence. Comparison 24 showed a statistically significant θ_D_ and relaxed selection, indicating functional divergence. (TIF 6617 kb)

## Abbreviations

ASDSF: A standard deviation of split frequencies
Branch-site REL: Random effects branch-site method
BY: Bayesian method
DLTRS: Duplications, losses, transfers, rates & sequence evolution
HyPhy: Hypothesis testing using phylogenies
Kb: Kilobases
MCMC: Metropolis-coupled Markov chain Monte Carlo
MEME: Mixed effects model of evolution
ML: Maximum likelihood
MSP3: Merozoite surface protein 3
MSP7: Merozoite surface protein 7
ORFs: Open reading frames
PvivTRAg56.2: *P. vivax* tryptophan-rich antigen 56.2
RDP: Recombination detection program
θ_D_: Coefficient of divergence
ω: Omega rate (d_N_/d_S_)
