# Supplementary material for: Evidence of functional divergence in MSP7 paralogous proteins: a molecular-evolutionary and phylogenetic analysis
Source: BMC Evol Biol. 2016 Nov 28;16:256. doi: 10.1186/s12862-016-0830-x (PMC5126858; doi:10.1186/s12862-016-0830-x)
Supplement: Additional file 2: — Putative donor/acceptor sites in P. vinckei vinckei msp7A. (PDF 42 kb) [file 12862_2016_830_MOESM2_ESM.pdf]

Evidence of functional divergence in MSP7 paralogous proteins: a molecular-evolutionary and phylogenetic analysis

Additional file 2. Putative donor/acceptor sites in *P. vinckei vinckei msp7A*.

```
                       *        20         *        40         *        60         *        80         *       100
PvinvA-DNA : ATGAAGGGAAAATATGCATTATTAGGTACCTTAGTTTTGTTGAATTGCGTATTAGGCAATAAAACTGATACAATAGAAGATGAGATAAATGAATTAACTGAAAAG :  105
PvinvA-RNA : ATGAAGGGAAAATATGCATTATTAGGTACCTTAGTTTTGTTGAATTGCGTATTAGGCAATAAAACTGATACAATAGAAGATGAGATAAATGAATTAACTGAAAAG :  105
             ATGAAGGGAAAATATGCATTATTAGGTACCTTAGTTTTGTTGAATTGCGTATTAGGCAATAAAACTGATACAATAGAAGATGAGATAAATGAATTAACTGAAAAG

                  *       120         *       140         *       160         *       180         *       200         *
PvinvA-DNA : TTAAACAATTTAGAACAAGATATCGCTTCTAATCACATCTCCGAAGATGAGGTTAGCGACGAAATCGAGGAATTAAAGATGAAGATCGAGGAGTTGAAAAAGCTG :  210
PvinvA-RNA : TTAAACAATTTAGAACAAGATATCGCTTCTAATCACATCTCCGAAGATGAGGTTAGCGACGAAATCGAGGAATTAAAGATGAAGATCGAGGAGTTGAAAAAGCTG :  210
             TTAAACAATTTAGAACAAGATATCGCTTCTAATCACATCTCCGAAGATGAGGTTAGCGACGAAATCGAGGAATTAAAGATGAAGATCGAGGAGTTGAAAAAGCTG

                    220         *       240         *       260         *       280         *       300         *
PvinvA-DNA : GCCGAAGATTATGACAATGACACAGTCGAAGGTAATGCAGAGACACAAGAGGGTGGAAGTGCAGGCACAGGCGGTGCAGATGGAGTTAGTGCAGGCGGGTCAGGT :  315
PvinvA-RNA : GCCGAAGATTATGACAATGACACAGTCGAAGGTAATGCAGAGACACAAGAGGGTGGAAGTGCAGGCACAGGCGGTGCAGATGGAGTTAGTGCAGGCGGGTCAGGT :  315
             GCCGAAGATTATGACAATGACACAGTCGAAGGTAATGCAGAGACACAAGAGGGTGGAAGTGCAGGCACAGGCGGTGCAGATGGAGTTAGTGCAGGCGGGTCAGGT

                320         *       340         *       360         *       380         *       400         *       420
PvinvA-DNA : TCTAAGGCAGCTGGAGATAATGCAGACGCAGGCGGTTCAGGTGTGGGTGGCGCAGGTGGCCCAGGTGCAGGCGGAAGCACAGGCGGTGCAAGCGGTTCAGGTTCT :  420
PvinvA-RNA : TCTAAGGCAGCTGGAGATAATGCAGACGCAGGCGGTTCAGGTGTGGGTGGCGCAGGTGGCCCAGGTGCAGGCGGAAGCACAGGCGGTGCAAGCGGTTCAGGTTCT :  420
             TCTAAGGCAGCTGGAGATAATGCAGACGCAGGCGGTTCAGGTGTGGGTGGCGCAGGTGGCCCAGGTGCAGGCGGAAGCACAGGCGGTGCAAGCGGTTCAGGTTCT

                       *       440         *       460         *       480         *       500         *       520
PvinvA-DNA : GGCGCAGACGCAGGCACAAGTGGAAGCGGTTCAGGTGTGGATGGCGCAGGTGCAGGCGCAAGTGGACCAACCGGACCAGCCGGACCAGGTAGTTCAACTGGGCCA :  525
PvinvA-RNA : GGCGCAGACGCAGGCACAAGTGGAAGCGGTTCAGGTGTGGATGGCGCAGGTGCAGGCGCAAGTGGACCAACCGGACCAGCCGGACCAGGTAGTTCAACTGGGCCA :  525
             GGCGCAGACGCAGGCACAAGTGGAAGCGGTTCAGGTGTGGATGGCGCAGGTGCAGGCGCAAGTGGACCAACCGGACCAGCCGGACCAGGTAGTTCAACTGGGCCA

                  *       540         *       560         *       580         *       600         *       620         *
PvinvA-DNA : AACGGAGCAAGTAGCCCAAATGCGCCAGGAGGTACTGGAGCTCAAGATAGTTCTAAGGGAACTGGAAGCCAAGGCGTTTCTCAGGAACCTGGAAGTCAAGGTGAT :  630
PvinvA-RNA : AACGGAGCAAGTAGCCCAAATGCGCCAGGAGGTACTGGAGCTCAAGATAGTTCTAAGGGAACTGGAAGCCAAGGC------------------------------ :  600
             AACGGAGCAAGTAGCCCAAATGCGCCAGGAGGTACTGGAGCTCAAGATAGTTCTAAGGGAACTGGAAGCCAAGGC

                    640         *       660         *       680         *       700         *       720         *
PvinvA-DNA : TCTAAGGGACCTGGAAGCCAAGGTGATTCTAAGGAAGCTGGAAGCCAAGATGGTTCTAAGGAATCCCAGGGAACTGATACAAAACAGAAATCTCAGGATTCTCAG :  735
PvinvA-RNA : --------------------------------------------------------------------------------------------------------- :    -

                740         *       760         *       780         *       800         *       820         *       840
PvinvA-DNA : GAATCCCAAGGAGCCAAGGACCCCGCTCCCGATGCAGAGCCAAAAGGGTCCGGAATAATATATCTTGATAGCCTTTACGATGAACTTCTTAGTGACGCGAATAAA :  840
```

```
PvinvA-RNA : --------------------------------------------------------------------------------------------------------- :    -

                     *        860         *        880         *        900         *        920         *        940
PvinvA-DNA : AAAAAATTAATCGATTCTAGCACCATACTAAATATAATGAATTAAAAAAAAAAATATGATCATTTTGCAATAACACCAAAAGAAGCTGAAATAATAAAAGATTTAT :  945
PvinvA-RNA : --------------------CACCATACTAAATATAATGAATTAAAAAAAAAAATATGATCATTTTGCAATAACACCAAAAGAAGCTGAAATAATAAAAGATTTAT :  685
                                 CACCATACTAAATATAATGAATTAAAAAAAAAAATATGATCATTTTGCAATAACACCAAAAGAAGCTGAAATAATAAAAGATTTAT

                 *        960         *        980         *       1000         *       1020         *       1040         *
PvinvA-DNA : TAGTAAAAATGTTTGTAACTAATACTGAAAATAAAGCAAATGAATTATTAGCTGTATTTAAAAAAGCTTTAACTGATGAAGAATTTGCAGAAGAATTCGATAATA : 1050
PvinvA-RNA : TAGTAAAAATGTTTGTAACTAATACTGAAAATAAAGCAAATGAATTATTAGCTGTATTTAAAAAAGCTTTAACTGATGAAGAATTTGCAGAAGAATTCGATAATA :  790
              TAGTAAAAATGTTTGTAACTAATACTGAAAATAAAGCAAATGAATTATTAGCTGTATTTAAAAAAGCTTTAACTGATGAAGAATTTGCAGAAGAATTCGATAATA

                    1060         *       1080         *       1100         *       1120         *       1140         *
PvinvA-DNA : TAATATCTGGTATTTATGCCTTTTCAAAAAAAAAATAATCATTTAGTAATTGACCAAGTAGAATATAAAGAAAAAATATGGTAAATTATATGAAACTATGAGCAAAC : 1155
PvinvA-RNA : TAATATCTGGTATTTATGCCTTTTCAAAAAAAAAATAATCATTTAGTAATTGACCAAGTAGAATATAAAGAAAAAATATGGTAAATTATATGAAACTATGAGCAAAC :  895
              TAATATCTGGTATTTATGCCTTTTCAAAAAAAAAATAATCATTTAGTAATTGACCAAGTAGAATATAAAGAAAAAATATGGTAAATTATATGAAACTATGAGCAAAC

               1160         *       1180         *
PvinvA-DNA : TTTTTCAAACTTCATCTTTTCAAGCATCAACCAATACTGCCTAA : 1199
PvinvA-RNA : TTTTTCAAACTTCATCTTTTCAAGCATCAACCAATACTGCCTAA :  939
              TTTTTCAAACTTCATCTTTTCAAGCATCAACCAATACTGCCTAA
```
