# Supplementary figures and images for: Evidence of functional divergence in MSP7 paralogous proteins: a molecular-evolutionary and phylogenetic analysis
Source: BMC Evol Biol. 2016 Nov 28;16:256. doi: 10.1186/s12862-016-0830-x (PMC5126858; doi:10.1186/s12862-016-0830-x)

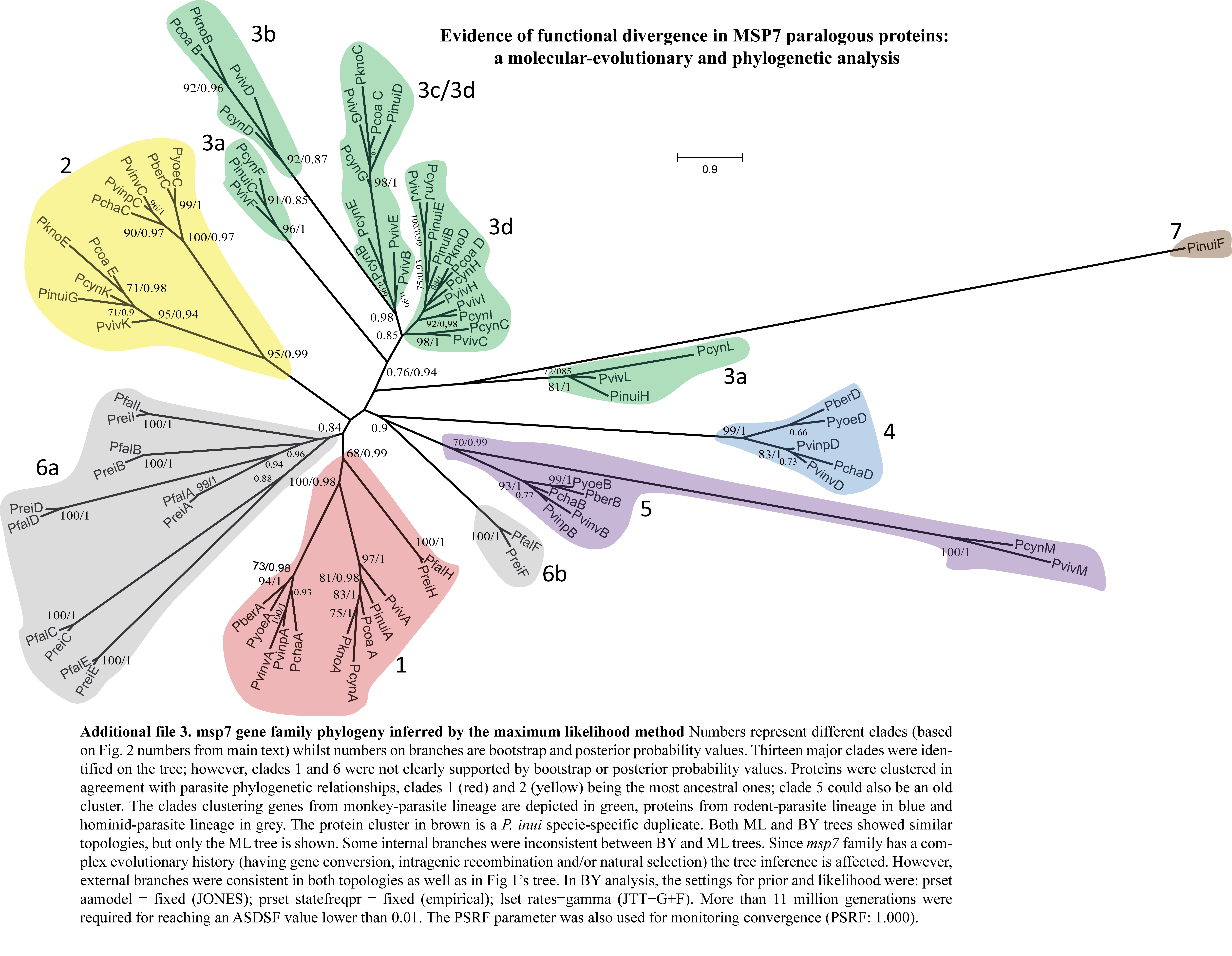

Supplement: Additional file 3: — msp7 gene family phylogeny inferred by the maximum likelihood method. Numbers represent different clades (based on Fig. 2 numbers from main text) whilst numbers on branches are bootstrap and posterior probability values. Thirteen major clades were identified on the tree; however, clades 1 and 6 were not clearly supported by bootstrap values. Proteins were clustered in agreement with parasite phylogenetic relationships, clades 1 (red) and 2 (yellow) being the most ancestral ones; clade 5 could also be an old cluster. The clades clustering genes from monkey-parasite lineage are depicted in green, proteins from rodent-parasite lineage in blue and hominid-parasite lineage in grey. The protein cluster in brown is a P. inui specie-specific duplicate. Both ML and BY trees showed similar topologies, but only the ML tree is shown. Some internal branches were inconsistent between BY and ML trees. Since msp7 family has a complex evolutionary history (having gene conversion, intragenic recombination and/or natural selection) the tree inference is affected. However, external branches were consistent in both topologies as well as in Fig 2’s tree. In BY analysis, the settings for prior and likelihood were: prset aamodel = fixed (JONES); prset statefreqpr = fixed (empirical); lset rates = gamma (JTT + G + F). More than 11 million generations were required for reaching an ASDSF value lower than 0.01. The PSRF parameter was also used for monitoring convergence (PSRF: 1.000). (TIF 3836 kb) [file 12862_2016_830_MOESM3_ESM.tif]

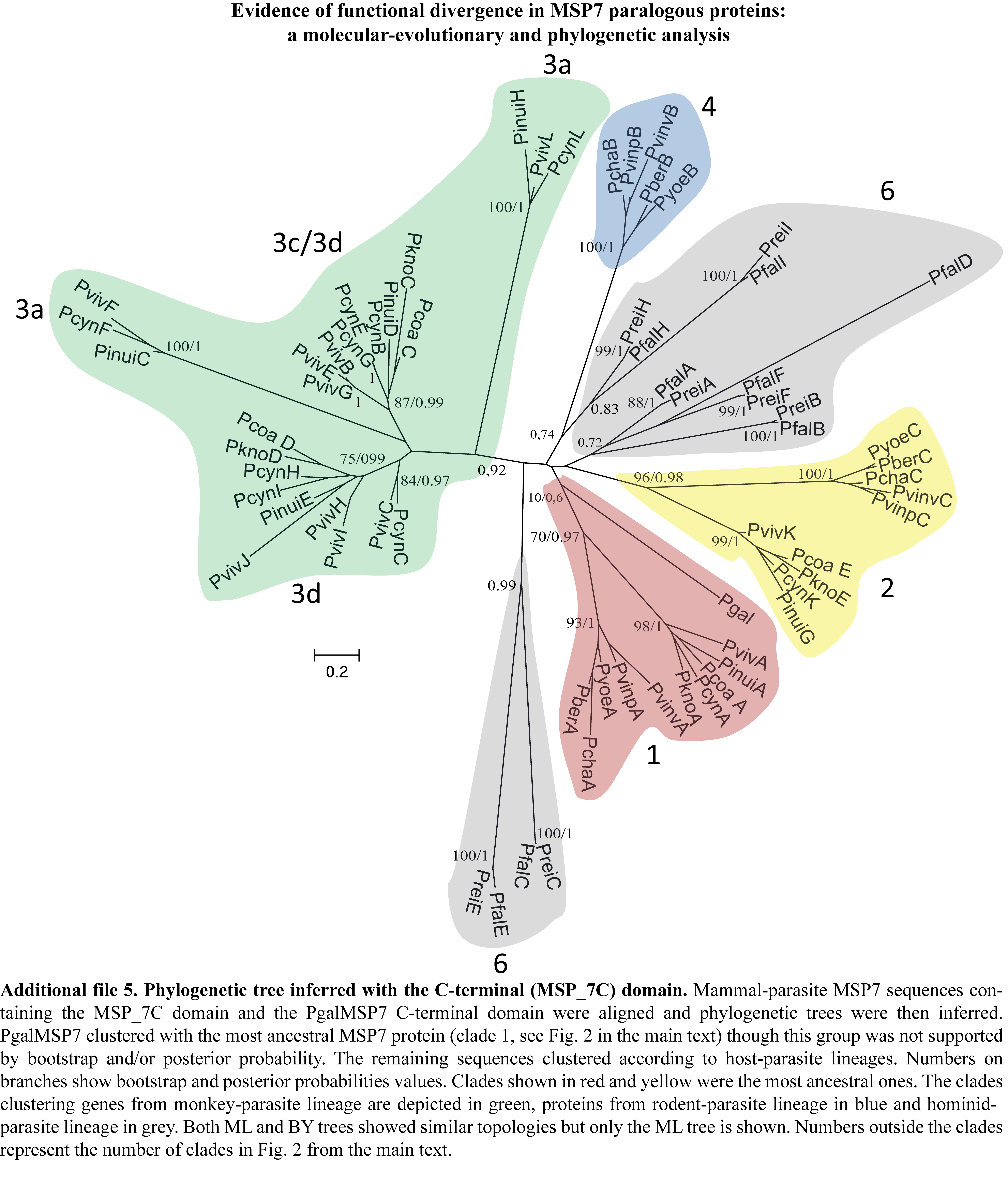

Supplement: Additional file 5: — Phylogenetic tree inferred with the C-terminal (MSP_7C) domain. Mammal-parasite MSP7 sequences containing the MSP_7C domain and the PgalMSP7 C-terminal domain were aligned and phylogenetic trees were then inferred. PgalMSP7 clustered with the most ancestral MSP7 protein (clade 1, see Fig. 2 in the main text) though this group was not supported by bootstrap and/or posterior probability. The remaining sequences clustered according to host-parasite lineages. Numbers on branches show bootstrap and posterior probabilities values. Clades shown in red and yellow were the most ancestral ones. The clades clustering genes from monkey-parasite lineage are depicted in green, proteins from rodent-parasite lineage in blue and hominid-parasite lineage in grey. Both ML and BY trees showed similar topologies but only the ML tree is shown. Numbers outside the clades represent the number of clades in Fig. 2 from the main text. (TIF 3038 kb) [file 12862_2016_830_MOESM5_ESM.tif]

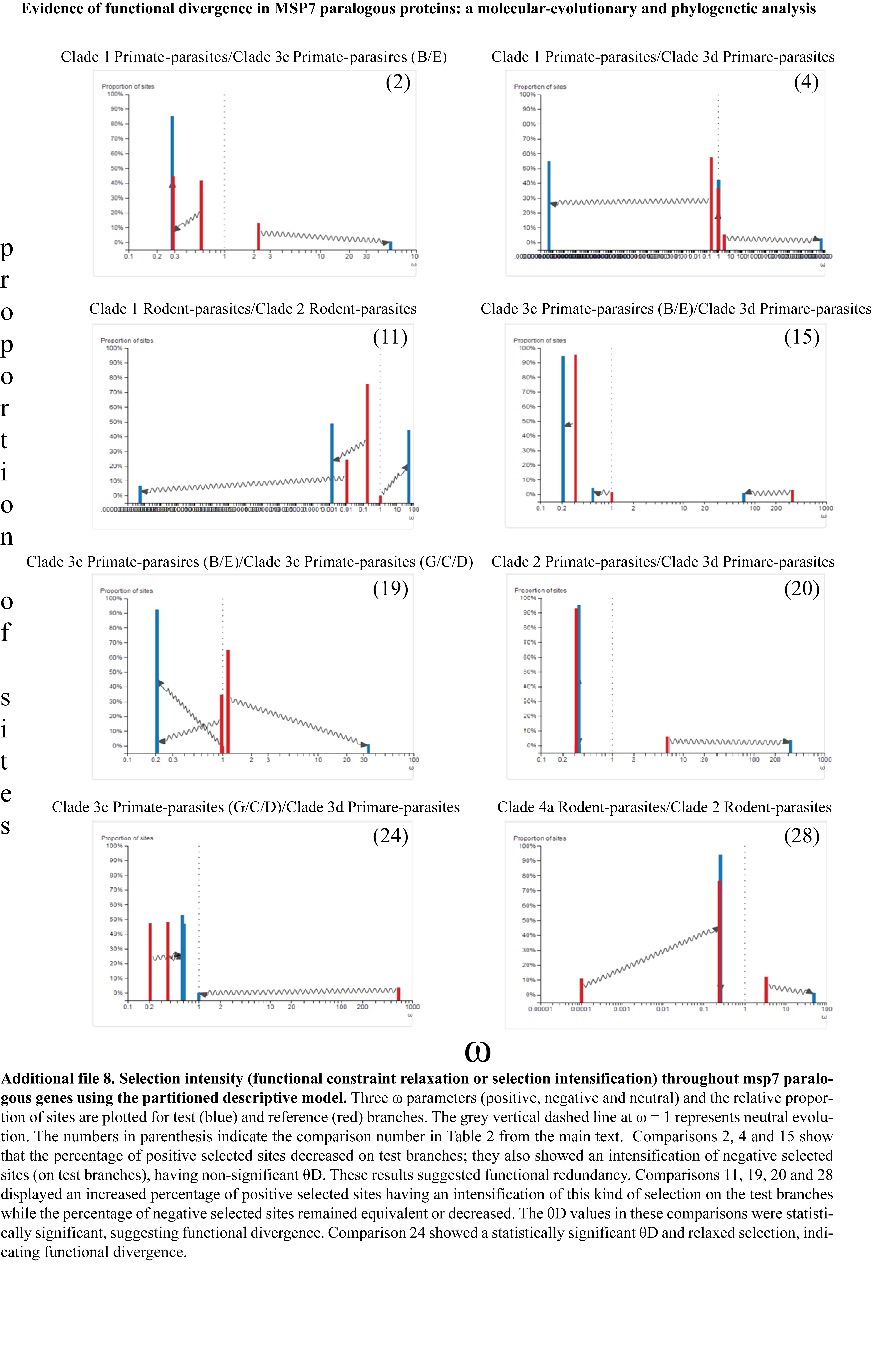

Supplement: Additional file 8: — Selection intensity (functional constraint relaxation or selection intensification) throughout msp7 paralogous genes using the partitioned descriptive model. Three ω parameters (positive, negative and neutral) and the relative proportion of sites are plotted for test (blue) and reference (red) branches. The grey vertical dashed line at ω = 1 represents neutral evolution. The numbers in parenthesis indicate the comparison number in Table 2 from the main text. Comparisons 2, 4 and 15 show that the percentage of positive selected sites decreased on test branches; they also showed an intensification of negative selected sites (on test branches), having non-significant θD. These results suggested functional redundancy. Comparisons 11, 19, 20 and 28 displayed an increased percentage of positive selected sites having an intensification of this kind of selection on the test branches whilst the percentage of negative selected sites remained equivalent or decreased. The θD values in these comparisons were statistically significant, suggesting functional divergence. Comparison 24 showed a statistically significant θD and relaxed selection, indicating functional divergence. (TIF 6617 kb) [file 12862_2016_830_MOESM8_ESM.tif]
