# Supplementary material for: Evidence of functional divergence in MSP7 paralogous proteins: a molecular-evolutionary and phylogenetic analysis
Source: BMC Evol Biol. 2016 Nov 28;16:256. doi: 10.1186/s12862-016-0830-x (PMC5126858; doi:10.1186/s12862-016-0830-x)
Supplement: Additional file 4: — Similarity values in the N-terminal region between MSP7 proteins and sequences lacking an MSP_7C domain. (PDF 24 kb) [file 12862_2016_830_MOESM4_ESM.pdf]

Evidence of functional divergence in MSP7 paralogous proteins: a molecular-evolutionary and phylogenetic analysis

Additional file 4. Similarity values in the N-terminal region between MSP7 proteins and sequences lacking an MSP_7C domain.

	PvivMSP7D
PvivMSP7B	49,8
PvivMSP7E	63,2

	PcynMSP7D
PcynMSP7B	48,8
PcynMSP7E	49,4

	PvivMSP7M
PvivMSP7A	53,2

	PcynMSP7M
PcynMSP7A	50,5

	PknoMSP7D
PknoMSP7B	56,2

	PinuMSP7F
PinuMSP7H	56,2

	PchaMSP7D
PchaMSP7C	42,1

	PvinpMSP7D
PvinpMSP7C	60,0

	PvinvMSP7D
PvinvMSP7C	57,5

	PberMSP7D
PberMSP7C	52,6

	PyoeMSP7D
PyoeMSP7C	62,6
