# Supplementary material for: Evidence of functional divergence in MSP7 paralogous proteins: a molecular-evolutionary and phylogenetic analysis
Source: BMC Evol Biol. 2016 Nov 28;16:256. doi: 10.1186/s12862-016-0830-x (PMC5126858; doi:10.1186/s12862-016-0830-x)
Supplement: Additional file 6: — Episodic positive selection on MSP7 branches. ω + values reflect the maximum likelihood estimate rate of positive selection. p-value obtained after Holm-Bonferroni multiple testing correction. The Branch-site REL method was performed by HyPhy software using both amino acid and DNA phylogenies. The Datamonkey web server was also used for calculating this method. The number of sites under episodic positive selection was identified by MEME using Datamonkey. The letters in the first panel correspond to the letters in Fig. 4 from the main text. (PDF 292 kb) [file 12862_2016_830_MOESM6_ESM.pdf]

Evidence of functional divergence in MSP7 paralogous proteins: a molecular-evolutionary and phylogenetic analysis

Additional file 6. Episodic positive selection on MSP7 branches.

		Phylogenetic tree inferred						Sites under episodic selection
		Amino acid alignment		DNA alignment		Datamonkey web server		
Letter from Fig. 4	*msp7* Branch	ω+	p-value	ω+	p-value	ω+	p-value	
A	Pinu/Pkno/Pcoa A	10000	0.0073	3362.94	5,06E-03	-	-	24
	Pkno/Pcoa A	-	-	-	-	200.62	0.009	
	PvinvA	196.69	2,98E-01	193.25	3,15E-01	281.41	0.0001	
	PvinpA	10000	0.00071	1207.83	0.0013	10000	0.000	
	PchaA	374.01	0.018	377.47	0.031	59.89	0.030	
	PinuiA	-	-	3050.5	0.042	-	-	
	Pviva	-	-	-	-	1574.57	0.0001	
	PcynA	-	-	-	-	33.77	0.014	
B	PcynB	1790.43	1,74E-08	1790.43	1,02E-06	1792.62	0.0001	12
	PcynE	1790.43	7,17E+00	1790.43	7,17E+00	3328.87	0.0001	
	PvivB	143.48	1,75E-06	143.48	1,75E-06	5836.17	0.0001	
	PvivE	132.53	0.019	132.53	0.019	3328.87	0.019	
C	PiniB_p	3111.99	1,79E-01	3111.99	1,79E-01	24.96	0.0001	-
D	PvivF	3333.11	0.0040	3333.11	0.0040	3333.51	0.004	5
E	Pkno/Pcoa C	327.92	0.019	-	-	-	-	42
	PcynG	10000	0.022	3334.11	0.048	-	-	
	PinuD	169.82	3,73E-07	260.74	2,84E-05	26.86	0.0001	
	PvivG/PinuD	10000	0.00069	-	-	-	-	
	PknoC	-	-	119.20	0.039	126.41	0.045	
F	Pkno/Pcoa D	399.40	0.00040	399.35	0.00040	38.51	0.001	46
	Pviv/Pcyn I	734.78	9,64E-05	734.45	9,66E-05	71.29	0.0001	

	**PvivI/PcynI/PinuE**	10000	1,69E-04	10000	1,69E-04	-	-	
	**PcoaD**	374.13	1,15E+00	374.82	1,15E+00	518.40	0.0001	
	**PknoD**	18.25	1,69E+00	182.49	1,69E+00	18.29	0.0001	
	**PvivH**	451.91	0.012	45.23	0.012	21.61	0.018	
	**PinuE**	10000	0.00022	10000	0.00022	262.81	0.000	
	**PcynI**	3333.58	0.039	3621.29	0.039	-	-	
G	**Pber/Pyoe C**	-	-	-	-	17.42	0.03	25
H	**PvivL**	134.41	0.012	134.18	0.012	13.54	0.012	7
I	**PvinvB**	663.74	0.00038	680.15	0.00017	-	0.01	6
	**PchaB**	482.01	0.0031	97.60	4,94E+00	-	0.0001	
	**PvinpB**	195.52	0.0019	268.56	0.00057	-	-	
	**PberB**	975.47	0.038	143.18	0.0086	-	-	

ω+ values reflect the maximum likelihood estimate rate of positive selection. p-value obtained after Holm-Bonferroni multiple testing correction. The Branch-site REL method was performed by HyPhy software by using both amino acid and DNA phylogenies. The Datamonkey web server also involved using this method. The number of sites under episodic positive selection was identified by MEME using Datamonkey. The letters in the first panel correspond to the letters in Fig. 4 from the main text.
