# Supplementary material for: Evidence of functional divergence in MSP7 paralogous proteins: a molecular-evolutionary and phylogenetic analysis
Source: BMC Evol Biol. 2016 Nov 28;16:256. doi: 10.1186/s12862-016-0830-x (PMC5126858; doi:10.1186/s12862-016-0830-x)
Supplement: Additional file 7: — Putative sites involved in functional divergence. DIVERGE computed a posterior probability for detecting putative amino acids responsible for functional divergence in pairwise comparisons having statistical significant θD values. Such putative sites were highlighted in green and sites in the C-terminal region (MSP_7C domain) under positive selection found by the MEME method were tagged with a red plus symbol (+). Since functional divergence could involve relaxation of functional constraint or could be due to positive selection, a perfect correlation between putative amino acids responsible for divergence and positive selection would not have been expected. Positive selection might be involved in the acquisition of a new role but could also be the outcome of adaptation to a new host during Plasmodium’s evolutionary history. Positive selected sites were inferred using the clade (e.g. sequences within clade 1) but not using the sequences from the comparison (e.g. Clade 1 Primate-parasites vs Clade 2 Primate-parasites). (PDF 478 kb) [file 12862_2016_830_MOESM7_ESM.pdf]

Evidence of functional divergence in MSP7 paralogous proteins: a molecular-evolutionary and phylogenetic analysis

Additional file 7. (.pdf). Putative sites involved in functional divergence.

Comparison 1: Clade 1 Primate-parasites vs Clade 1 Rodent-parasites

```
                 *    +    20    +    + *         40         *        60  + +   *      80
PvivA  : VKHVDTLCDELLAGENKKNVLDEGEDHSQYNIFRKQYDKMVLNKTEYNISLK-LDTMLTNGQVEREKKNTLIKTFKKALYDKQYSE :  85
PcynA  : VNHVDTLFDELLAEDNKKHMMDEGEHHSAYNNLRKQYDELSLNKTEYAISLKLLDTMLSSEKVGEKRKNALVEMFKKAMYDKEYSE :  86
PinuiA : VNHVDILYDKLLEGADKRNIMDKGEHHTKYNNFRQQYDHLILNQTEYDISFKLLDTMLSSGKDGDARKNALFETFKKAMYDEQYSE :  86
Pcoa_A : LHHLDLLYDELLAGDNQKNMMDEGQYHSKYNNFRKKYDQLILNQTEYDVSLKLLDKMLSSGKVGEEKINALIETFKKAMDDEEYGE :  86
PknoA  : IHHFDLLYDNLLAGENKKNMMDEGEHHSAYNNFRKQYDQLILSQTEYDICLK-LDTMLSNGMVEEAKKNALKETFKKAMYDKDYGE :  85
PberA  : IKYLDTIYDEILTNSENKNLINDNENHSKYREFKKKYDNFAITPKESEIIKD-LIKMFVTN--SNNKLNELLAVFKKALHDNKFAE :  83
PyoeA  : IKYLDTIYDELLTNSENKNLINNNENHNKYSEFKKRYDNFALTPKESEIIKD-LIKMFVTN--SDNKLNELLVVFKKALHDKEFAE :  83
PvinvA : --------------QDSSKGTGSQGHHTKYNELKKKYDHFAITPKEAEIIKDLLVKMFVTN--TENKANELLAVFKKALTDEEFAE :  70
PchaA  : IKYLDTLYDELLTDDKNQNLID-AENHSKYNEFKKKYDKFAITPKEAEIIKD-LVKMFVTN--PDNKVKELLVVFKKALHDKEFAE :  82
PvinpA : --YLDTLYDELLTDAKKNSLID-SELHSKYNEFKKKYDKFAITPKEAEIIKDLLVKMFVTN--IESKTNELLAVFKKALHDKEFAE :  81
             d   d  l               H  Yn f4k YD   6   E  6     L  M           4 n L   FKKA6 D  5 E

            *        100       +     *     + +120             *
PvivA  : KLRNLISGVYAFAKRNNFIDGDKVKEGD-YSKLFEYIGCM---- : 124
PcynA  : KFKSLIYGVYLFAKRHNFLDEGKVKGED-YNKLFNYVGNL---- : 125
PinuiA : KFKNLISGVYGFAKRNNFLDMERMNEES-YKKLFDYIGSL---- : 125
Pcoa_A : KFKNLISGVYGFAKRNNFLDGSKMNEEN-YNKLFDYIGSL---- : 125
PknoA  : KFKNLISGVYAFAKRNNFLDENKVKGQD-YNKLFDYICSL---- : 124
PberA  : ELNNLISGIYAFSKRHNYLVTEKEEYKEKYEKLYENISKM---- : 123
PyoeA  : ELNNLISGIYAFSKRHNYLVTEKDEYREKYEKLYENISKV---- : 123
PvinvA : EFDNIISGIYAFSKKNNHLVIDQVEYKEKYGKLYETMSKL---- : 110
PchaA  : EFNNLISGIYAFSKRNNHLVIDQKEYKDKYNKLYEHISNL---- : 122
PvinpA : EFNNLISGIYAFSKKYNHLVIDQVEYKEKYDKLYE--------- : 116
            n6IsG6Y  F  K4  N  6          Y  KL5
```

Comparison 3: Clade 1 Rodent-parasites vs Clade 3d Primate-parasites (B/E)

```
                  *    +   20   +    +*        40         *        60   + +   *        80
PcynB  : IKYMDKLYDDILSTLGKTNEIHIPLYHSKYNTIRKDYE-LSMKPVEYQIVKNLFNVGFKKE-GESSAANSLTEVFKKVLVDEKFQD :  84
PcynE  : IKYMDKLYDDILSTSGKTNEIHIPLYHSKYNTIRKDYE-LSMKPVEYQIVKNLFNVGFKKE-GESSAANSLTEVFKKVLVDEKFQD :  84
PvivB  : IKYLDKLYDEVLTTSDNTSGIHVPDYHSKYNTIRQKYE-YSMNPVEYEIVKN-FNVGFKNDGAASSDATPLVDVFKKALADEKFQA :  84
PvivE  : IKYLDKLYDEVLTTSDNTNGIHVPDYHSKYNTIRQKYE-YSMNPVEYEIVKN-FNVGFKNDGAASSDATPLVDVFKKALADETFQA :  84
PberA  : IKYLDTIYDEILTNSENKNLINDNENHSKYREFKKKYDNFAITPKESEIIKD-LIKMFVTN--SNNKLNELLAVFKKALHDNKFAE :  83
PyoeA  : IKYLDTIYDELLTNSENKNLINNNENHNKYSEFKKRYDNFALTPKESEIIKD-LTKMFVTN--SDNKLNELLVVFKKALHDKEFAE :  83
PvinvA : --------------QDSSKGTGSQGHHTKYNELKKKYDHFAITPKEAEIIKDLLVKMFVTN--TENKANELLAVFKKALTDEEFAE :  70
PchaA  : IKYLDTLYDELLTDDKNQNLID-AENHSKYNEFKKKYDKFAITPKEAEIIKD-LVKMFVTN--PDNKVKELLVVFKKALHDKEFAE :  82
PvinpA : --YLDTLYDELLTDAKKNSLID-SELHSKYNEFKKKYDKFAITPKEAEIIKDLLVKMFVTN--IESKTNELLAVFKKALHDKEFAE :  81
         iky d  yd  l        i     H KYn  4k Y    6 P E 2I6K1     F            L  VFKKaL D  F

            *       100    +    *    + +120         *
PcynB  : EFNNFVHGLYGFAKRHNYLGKERLENTTSDSDLLKNAFSL---- : 124
PcynE  : EFNNFVHGLYGFAKRHNYLGNKRLENTTVDSDLLKNAFSL---- : 124
PvivB  : EFDNFVHGLYGFAKRHSYLSEARMKDNKLYSDLLKNAISL---- : 124
PvivE  : EFDNFVHGLYGFAKRHNYLSEARMKDADRYTNLLKNAISL---- : 124
PberA  : ELNNLISGIYAFSKRHNYLVTEKEEYKEKYEKLYENISKM---- : 123
PyoeA  : ELNNLISGIYAFSKRHNYLVTEKDEYREKYEKLYENISKV---- : 123
PvinvA : EFDNIISGIYAFSKKNNHLVIDQVEYKEKYGKLYETMSKL---- : 110
PchaA  : EFNNLISGIYAFSKRNNHLVIDQKEYKDKYNKLYEHISNL---- : 122
PvinpA : EFNNLISGIYAFSKKYNHLVIDQVEYKEKYDKLYE--------- : 116
         Ef1N 6 G6Y F K4 n L     e    y  L
```

Comparison 5: Clade 1 Primate-parasites vs Clade 3c Primate-parasites (G/C/D)

```
                                      +                            +
                  *    +  +20  +++  ++*        40         *  + +   60   + +++ * +      80
PvivA  : VKHVDTLCDELLAGENKKNVLDEGEDHSQYNIFRKQYDKMVLNKTEYNISLK-LDTMLTNGQVEREKKNTLIKTFKKALYDKQYSE :  85
PcynA  : VNHVDTLFDELLAEDNKKHMMDEGEHHSAYNNLRKQYDELSLNKTEYAISLKLLDTMLSSEKVGEKRKNALVEMFKKAMYDKEYSE :  86
PinuiA : VNHVDILYDKLLEGADKRNIMDKGEHHTKYNNFRQQYDHLILNQTEYDISFKLLDTMLSSGKDGDARKNALEETFKKAMYDEQYSE :  86
PcoaA  : LHHLDLLYDELLAGDNQKNMMDEGQYHSKYNNFRKKYDQLILNQTEYDVSLKLLDKMLSSGKVGEEKINALIETFKKAMDDEEYGE :  86
PknoA  : IHHFDLLYDNLLAGENKKNMMDEGEHHSAYNNFRKQYDQLILSQTEYDICLK-LDTMLSNGMVEEAKKNALKETFKKAMYDKDYGE :  85
PvivG  : IKYLDKLYDEVLTTSDNTSGIHVPDYHSKYNTIRQKYE-YSMNPVEYEIVKN-FNVGFKNDGAASSDATPLVDVFKKALADEKFQA :  84
PcynG  : IKYMDKLYDDILSTSGNTNEIHIPLYHSKYNTIRKDYE-LSMKPVEYQIVKNLFNVGFKKEGESSAA-NSLTEVFKKVLVDEKFQD :  84
PinuiD : IKYLDELYDEIMNISDRTNDINKQAYDSKYSTIKKKYK-LSMNPVEYQIVKNLFNVGFKKEGETSVT-TSLSEVFKKVLDDEKSQE :  84
PknoC  : INYLDELYDEIITTPDIKKKMQNSSYKTK-------YK-LSLNPAEYQIVKK-FELGFGNENESSAG-TSLVEVFKKVLDDEKFQK :  76
PcoaC  : ITFLDKLYDDIINTSDSKNEIHNTPYHTKYNAIRNKYE-LSMNPVEYQIVKNLFEVGFNKEGESSAD-TSFIDIFKKVLDDEKFQD :  84
         6   D L D 66        6     h3 yn     Y    6n  EY 6                     l   FKK 6 D

            *       100    +    *    + +120         *
PvivA  : KLRNLISGVYAFAKRNNFIDGDKVKEGD-YSKLFEYIGCM---- : 124
PcynA  : KFKSLIYGVYLFAKRHNFLDEGKVKGED-YNKLFNYVGNL---- : 125
PinuiA : KFKNLISGVYGFAKRNNFLDMERMNEES-YKKLFDYIGSL---- : 125
PcoaA  : KFKNLISGVYGFAKRNNFLDGSKMNDEN-YNKLFDYIGSL---- : 125
PknoA  : KFKNLISGVYAFAKRNNFLDENKVKGQD-YNKLFDYICSL---- : 124
PvivG  : EFDNFVHGLYGFAKRHSYLSKERMGDTTRYSDLLKNAISL---- : 124
PcynG  : EFNNFVHGLYGFAKRHNYLGKERLENTTSDSDLLKNAFSL---- : 124
PinuiD : QFDNFVEGLYGFAKRHSYLGKDGMDNTR-YSDLLKNAISL---- : 123
PknoC  : EFDNIVQGFYGFAKRHNYISQEQMNNTS-HTNLLKNAINL---- : 115
PcoaC  : EFDNFVQGLYGFAKRHNYLGKERMDNTTSYSNLLKNALSL---- : 124
          f n 6 G Y FAKR n56    6     y  L      6
```

Comparison 6: Clade 1 Rodent-parasites vs Clade 3c Primate-parasites (G/C/D)

```
                                     +                            +
         *    +  +20  +++  ++*        40         *  + +   60   + +++ * +      80
PvivG  : IKYLDKLYDEVLTTSDNTSGIHVPDYHSKYNTIRQKYE-YSMNPVEYEIVKN-FNVGFKNDGAASSDATPLVDVFKKALADEKFQA :  84
PcynG  : IKYMDKLYDDILSTSGNTNEIHIPLYHSKYNTIRKDYE-LSMKPVEYQIVKNLFNVGFKKEGESSAA-NSLTEVFKKVLVDEKFQD :  84
PinuiD : IKYLDELYDEIMNISDRTNDINKQAYDSKYSTIKKKYK-LSMNPVEYQIVKNLFNVGFKKEGETSVT-TSLSEVFKKVLDDEKSQE :  84
PknoC  : INYLDELYDEIITTPDIKKKMQNSSYKTK-------YK-LSLNPAEYQIVKK-FELGFGNENESSAG-TSLVEVFKKVLDDEKFQK :  76
PcoaC  : ITFLDKLYDDIINTSDSKNEIHNTPYHTKYNAIRNKYE-LSMNPVEYQIVKNLFEVGFNKEGESSAD-TSFIDIFKKVLDDEKFQD :  84
PberA  : IKYLDTIYDEILTNSENKNLINDNENHSKYREFKKKYDNFAITPKESEIIKD-LIKMFVTNSNNKL--NELLAVFKKALHDNKFAE :  83
PyoeA  : IKYLDTIYDELLTNSENKNLINNNENHNKYSEFKKRYDNFALTPKESEIIKD-LTKMFVTNSDNKL--NELLVVFKKALHDKEFAE :  83
PchaA  : IKYLDTLYDELLTDDKNQNLID-AENHSKYNEFKKKYDKFAITPKEAEIIKD-LVKMFVTNPDNKV--KELLVVFKKALHDKEFAE :  82
PvinvA : --------------QDSSKGTGSQGHHTKYNELKKKYDHFAITPKEAEIIKDLLVKMFVTNTENKA--NELLAVFKKALTDEEFAE :  70
PvinpA : --YLDTLYDELLTDAKKNSLID-SELHSKYNEFKKKYDKFAITPKEAEIIKDLLVKMFVTNIESKT--NELLAVFKKALHDKEFAE :  81
         i   d  yd                 h Ky      Y    6 P E 2I6K      F            l  6FKK L D  f

            *       100    +    *    + +120         *
PvivG  : EFDNFVHGLYGFAKRHSYLSKERMGDTTRYSDLLKNAISL---- : 124
PcynG  : EFNNFVHGLYGFAKRHNYLGKERLENTTSDSDLLKNAFSL---- : 124
PinuiD : QFDNFVEGLYGFAKRHSYLGKDGMDNTR-YSDLLKNAISL---- : 123
PknoC  : EFDNIVQGFYGFAKRHNYISQEQMNNTS-HTNLLKNAINL---- : 115
PcoaC  : EFDNFVQGLYGFAKRHNYLGKERMDNTTSYSNLLKNALSL---- : 124
PberA  : ELNNLISGIYAFSKRHNYLVTEKEEYKEKYEKLYENISKM---- : 123
PyoeA  : ELNNLISGIYAFSKRHNYLVTEKDEYREKYEKLYENISKV---- : 123
PchaA  : EFNNLISGIYAFSKRNNHLVIDQKEYKDKYNKLYEHISNL---- : 122
PvinvA : EFDNIISGIYAFSKKNNHLVIDQVEYKEKYGKLYETMSKL---- : 110
PvinpA : EFNNLISGIYAFSKKYNHLVIDQVEYKEKYDKLYE--------- : 116
         2f1N 6 G Y F K4 n 6          y  L
```

Comparison 7: Clade 1 Rodent-parasites vs Clade 3d Primate-parasites (C/H/E/D/I)

```
                       *        20        *        40         *        60          *        80
PberA  : IKYLDTIYDEILTNSENKNLINDNENHSKYREFKKKYDNFAITPKESEIIKD-LIKMFVTNS--NNKLNELLAVFKKALHDNKF :  81
PyoeA  : IKYLDTIYDELLTNSENKNLINNNENHNKYSEFKKRYDNFALTPKESEIIKD-LTKMFVTNS--DNKLNELLVVFKKALHDKEF :  81
PchaA  : IKYLDTLYDELLTDDKNQNLID-AENHSKYNEFKKKYDKFAITPKEAEIIKD-LVKMFVTNP--DNKVKELLVVFKKALHDKEF :  80
PvinvA : --------------QDSSKGTGSQGHHTKYNELKKKYDHFAITPKEAEIIKDLLVKMFVTNT--ENKANELLAVFKKALTDEEF :  68
PvinpA : --YLDTLYDELLTDAKKNSLID-SELHSKYNEFKKKYDKFAITPKEAEIIKDLLVKMFVTNI--ESKTNELLAVFKKALHDKEF :  79
PvivC  : VKYLDKLYDDVLATQDSANGIDVPPFHSKYNDFRKKYE-FKMNDSEYKIMKN-FDVSFKKEG-QQSSAACLVNVFKKVLDDEHL :  81
PcynC  : VKYLDKLYDEVLETPNSVNAIHTQPFHSKYNEFRKKYE-FTMNEREYQIVKNLFDVCFKKEG-EQSSTSCIVNMFKKVLDDKNF :  82
PknoD  : VKYLDKLYDEVLKGEDGKNGIHIPEFHSKYNDFRKKYE-LTMNEQEYQMMKK-FDAFFKKGE--STNAVCPLEFFKKVLNNMSL :  80
PcoaD  : VKYLDKLYDEVLNTENANNGIHVHPYHSKYNDFRKNYE-FTMNEQEYQIMKKLFDVFFKKEE--GSNSVCPIAFFKKVLDDLSL :  81
PvivH  : VKYLDKLYDEVLKTTDAKDEIHVPPFHSKYNDFRKKYE-FTMNEREYQIVKN-FDAFFKKDG--NPSPADAVSFFKKMLNDPNV :  80
PcynH  : VKYLDKLYDEVLNTSEEKNAIHVPAFHSKYNDFRTKYE-FTMNEQEYHIVKKLFDAFFKKEQ--NSSYDAPIKFFKNVLNDAEF :  81
PinuiE : VKYLDKLYDELLKTTEGKDGIHVPLFHSKYNDFRKKYE-HTMNEREYQIVKNLFEGFFKEGH---SKCGCPVEFFKKLLNDAGL :  80
PvivI  : VKYLDKLYDEVLKTTDAKDEIHVPPFHSKYNDFRKKYE-FTMNEREYQIVKN-FDAFFKKDG--NPSPADAVSFFKKMLNDPNV :  80
PcynI  : VKYLDKLYDEILKTTDVKDGIHVPAFHSKYNDFRTKYE-FTMNEREYQIVKNLFDTFFKKDG--NSNDDAPIKFFKNVLNDADF :  81
           kyld  yde l           i     H KYn f4k Y   f 6    E   66K       F                6   FKk L 1

                    *         100         *          120         *
PberA  : AEELNNLISGIYAFSKRHNYLVTEKEEYKEKYEKLYENISKM---- : 123
PyoeA  : AEELNNLISGIYAFSKRHNYLVTEKDEYREKYEKLYENISKV---- : 123
PchaA  : AEEFNNLISGIYAFSKRNNHLVIDQKEYKDKYNKLYEHISNL---- : 122
PvinvA : AEEFDNIISGIYAFSKKNNHLVIDQVEYKEKYGKLYETMSKL---- : 110
PvinpA : AEEFNNLISGIYAFSKKYNHLVIDQVEYKEKYDKLYE--------- : 116
PvivC  : QKEFDNFVHGFYGFAKRHNYLRGERMANENLYKDIEKNVVNL---- : 123
PcynC  : QKKFDNFVQGFYGFAKRHNYLRGERMANENLYKDILKNVVNL---- : 124
PknoD  : QEEFDNFQHGLYGFAKRHNYLRGEKTTNEKLYHDLLKNIINL---- : 122
PcoaD  : QKQFDNFQKGLYGFTKRHNYLRGEKTTNGQLYDELLKNIINL---- : 123
PvivH  : QKEFDNFVHGLYGFAKRHNYLRGERMTDTKLYDELLKNVVNL---- : 122
PcynH  : QKQFDNFQHGLYGFAKRHNYLRGDRMTDGQLYSELLKNVINL---- : 123
PinuiE : QKQFDNFMHGLYGFAKRHNYLRGERMTDRKLCEEVLKNVINL---- : 122
PvivI  : QKEFDNFVHGLYGFAKRHNYLRDERMNLTDAHKSLFVNTLSL---- : 122
PcynI  : QKQFDNFQHGLYGFAKRHNYLRGQRMNLTDAHKNLLLNALSL---- : 123
            f1N    G Y  F  K4hNyL           y  6  n
```

Comparison 10: Clade 1 Rodent-parasites vs Clade 2 Primate-parasites

```
                 *     +   20     +    +*         40          *    +    60    + +   *         80
PberA  : IKYLDTIYDEILTNSENKNLINDNENHSKYREFKKKYDNFA-ITPKESEIIKD-LIKMFVTNSN-N-KLNELLAVFKKALHDNKFA :  82
PyoeA  : IKYLDTIYDELLTNSENKNLINNNENHNKYSEFKKRYDNFA-LTPKESEIIKD-LTKMFVTNSD-N-KLNELLVVFKKALHDKEFA :  82
PchaA  : IKYLDTLYDELLTDDKNQNLID-AENHSKYNEFKKKYDKFA-ITPKEAEIIKD-LVKMFVTNPD-N-KVKELLVVFKKALHDKEFA :  81
PvinvA : --------------QDSSKGTGSQGHHTKYNELKKKYDHFA-ITPKEAEIIKDLLVKMFVTNTE-N-KANELLAVFKKALTDEEFA :  69
PvinpA : --YLDTLYDELLTDAKKNSLID-SELHSKYNEFKKKYDKFA-ITPKEAEIIKDLLVKMFVTN--IESKTNELLAVFKKALHDKEFA :  80
PvivK  : LKYSDELYEDILTSLNKKGCEEGTENNNKYNEFKKEYEMFISLNKEEYEIIEK-VDAFSMYNDSIDEDADSVYEAIKKSFTDPKFR :  85
PcynK  : LKYSDELYEDILMNLHKKGGKEGTDYNDKYNEFKKEYDMFISLNKDEYEIISKLVNAFSMYNDAIDEDADTVYEAIKKSFTDPKFK :  86
PinuiG : -KYSEKVYEDILISLNKRGGEQGTNENHKYNEFKKEYDMFISLNKDEYEIIEKLVDAFCVNNQPFDEDADSVYEAIKKSFTDPKFK :  85
PknoE  : LNYADDLYEDILSSLSKKGGEEGTNYDDKYNEFKKEYDRFISLSKDEYEIIGK-IDAFSMYNDAISEDTDSVYQAIKKSFTDKKFK :  85
PcoaE  : LDYADELYEEILSSLSKKDGEEGTNYDDKYNDFKKEYDMFISLSKDEYEIIGKLIDAFSMYNDGINEDADSVYEAIKKSFTDPKFK :  86
            y d  y   l                  KYnefKK Yd F  6    E EII     6     6 N            6     KK    D  F

             *          100     +      *    + +120          *
PberA  : EELNNLISGIYAFSKRHNYLVTEKEEYKEKYEKLYENISKM---- : 123
PyoeA  : EELNNLISGIYAFSKRHNYLVTEKDEYREKYEKLYENISKV---- : 123
PchaA  : EEFNNLISGIYAFSKRNNHLVIDQKEYKDKYNKLYEHISNL---- : 122
PvinvA : EEFDNIISGIYAFSKKNNHLVIDQVEYKEKYGKLYETMSKL---- : 110
PvinpA : EEFNNLISGIYAFSKKYNHLVIDQVEYKEKYDKLYE--------- : 116
PvivK  : KEFKDFMNGIYAYAKRKHYIRGTQTEKAKTYLTLFENVINL---- : 126
PcynK  : QQFGDFMNGIYAYASKKHNIRGAQTEEVKTYLMLFQNVINL---- : 127
PinuiG : KEFGDFINGIYDYSQKKHNIRGTQTEQTKTYHMLFQNVINF---- : 126
PknoE  : QEFKDFMNGIYTYTQKKHNIRGSQTEDIKKYLMLFQNIINYL--- : 127
PcoaE  : QEFKDFMNGIYSYAERKHHIRGAQTEQAKTYLMLFQNVINL---- : 127
          2f 1 6 GIY 5   4    6    q E     Y  L52
```

Comparison 11: Clade 1 Rodent-parasites vs Clade 2 Rodent-parasites

```
                  *    +   20   +    +*        40         *   +    60   + +   *        80
PberA  : IKYLDTIYDEILTNSENKNLINDNENHSKYREFKKKYDNFA-ITPKESEIIKD-LIKMFVTNSN-N-KLNELLAVFKKALHDNKFA :  82
PyoeA  : IKYLDTIYDELLTNSENKNLINNNENHNKYSEFKKRYDNFA-LTPKESEIIKD-LTKMFVTNSD-N-KLNELLVVFKKALHDKEFA :  82
PchaA  : IKYLDTLYDELLTDDKNQNLID-AENHSKYNEFKKKYDKFA-ITPKEAEIIKD-LVKMFVTNPD-N-KVKELLVVFKKALHDKEFA :  81
PvinvA : --------------QDSSKGTGSQGHHTKYNELKKKYDHFA-ITPKEAEIIKDLLVKMFVTNTE-N-KANELLAVFKKALTDEEFA :  69
PvinpA : --YLDTLYDELLTDAKKNSLID-SELHSKYNEFKKKYDKFA-ITPKEAEIIKDLLVKMFVTN--IESKTNELLAVFKKALHDKEFA :  80
PberC  : -NYTNDFYNAVIASLNEND--KNGNYKNKYEDFKNKYDNVIAINEKEFEIFSK-VEVFMKNHHDFNLNSNFLYELLTKALSDQNFK :  82
PyoeC  : -NYTNDFYNAVIANLNEND--KNGNYKNKYEDFKNKYDNVIAIDEKEFEIFSKLVEVFMKNHQDFSFNSNFLYELLTKALSDQGFK :  83
PchaC  : QNYTDDFFNSVIANINEND---NGDYKDKYEDFKSKYDNIIAINQKEFEIFSK-IEGFMQSNKDMNLSSDYLYEVLTKALSDKAFK :  82
PvinvC : -NYADDIFGSVIANINTSD---NGDYKVKYEDFKNKYDNLIAIDQTEFEIFSKLVEGFMKTNKEINLSSDYLYDVLTKTLSDKAFK :  82
PvinpC : -NYTDDFFNSVIANINTSD---NGEYKIKYEDFKNKYDNIMAIDQREFEIFSKLVEGFMKNNKEMNLSSDYLYEVITKALSDTAFK :  82
           y                         KY  fK 4YD    6   E EI    6      n   n     L  6  KaL D  F

            *       100    +    *    + +120         *
PberA  : EELNNLISGIYAFSKRHNYLVTEKEEYKEKYEKLYENISKM---- : 123
PyoeA  : EELNNLISGIYAFSKRHNYLVTEKDEYREKYEKLYENISKV---- : 123
PchaA  : EEFNNLISGIYAFSKRNNHLVIDQKEYKDKYNKLYEHISNL---- : 122
PvinvA : EEFDNIISGIYAFSKKNNHLVIDQVEYKEKYGKLYETMSKL---- : 110
PvinpA : EEFNNLISGIYAFSKKYNHLVIDQVEYKEKYDKLYE--------- : 116
PberC  : TEFKEFMNNMYNFVKKNQEGKVMTDNDKM-YMALFENVLSL---- : 122
PyoeC  : TEFKEFMNNMYNFVKKKQEGKPMTENDKI-YMTLFENVLSL---- : 123
PchaC  : AEFKEFMNSMYNFIKKKHEGKTLSENDKQ-YMALFENVLTL---- : 122
PvinvC : DEFKSFMNNMYNFVKKKHEGKLTTETDKQ-YMALFENVLSL---- : 122
PvinpC : AEFKDFMNNMYSFVKKKHEGKTLSESDKQ-YMTLFENVLSL---- : 122
          Ef   6  6Y F K4           4  Y  L5E
```

Comparison 12: Clade 2 Primate-parasites vs Clade 2 Rodent-parasites

```
                       *        20         *        40         *  +   60         *        80
PvivK  : LKYSDELYEDILTSLNKKGCEEGTENNNKYNEFKKEYEMFISLNKEEYEIIEK-VDAFSMYNDSIDEDADSVYEAIKKSFTDPKFR :  85
PcynK  : LKYSDELYEDILMNLHKKGGKEGTDYNDKYNEFKKEYDMFISLNKDEYEIISKLVNAFSMYNDAIDEDADTVYEAIKKSFTDPKFK :  86
PinuiG : -KYSEKVYEDILISLNKRGGEQGTNENHKYNEFKKEYDMFISLNKDEYEIIEKLVDAFCVNNQPFDEDADSVYEAIKKSFTDPKFK :  85
PknoE  : LNYADDLYEDILSSLSKKGGEEGTNYDDKYNEFKKEYDRFISLSKDEYEIIGK-IDAFSMYNDAISEDTDSVYQAIKKSFTDKKFK :  85
PcoaE  : LDYADELYEEILSSLSKKDGEEGTNYDDKYNDFKKEYDMFISLSKDEYEIIGKLIDAFSMYNDGINEDADSVYEAIKKSFTDPKFK :  86
PberC  : -NYTNDFYNAVIASLNEND--KNGNYKNKYEDFKNKYDNVIAINEKEFEIFSK-VEVFMKNHHDFNLNSNFLYELLTKALSDQNFK :  82
PyoeC  : -NYTNDFYNAVIANLNEND--KNGNYKNKYEDFKNKYDNVIAIDEKEFEIFSKLVEVFMKNHQDFSFNSNFLYELLTKALSDQGFK :  83
PchaC  : QNYTDDFFNSVIANINEND---NGDYKDKYEDFKSKYDNIIAINQKEFEIFSK-IEGFMQSNKDMNLSSDYLYEVLTKALSDKAFK :  82
PvinvC : -NYADDIFGSVIANINTSD---NGDYKVKYEDFKNKYDNLIAIDQTEFEIFSKLVEGFMKTNKEINLSSDYLYDVLTKTLSDKAFK :  82
PvinpC : -NYTDDFFNSVIANINTSD---NGEYKIKYEDFKNKYDNIMAIDQREFEIFSKLVEGFMKNNKEMNLSSDYLYEVITKALSDTAFK :  82
           Y    5  66  6          y  KY  FK  Yd  6 6   E5EI  K 6  F   n       1 6Y  6 K  3D  F4

            *        100         *        120         *
PvivK  : KEFKDFMNGIYAYAKRKHYIRGTQTEKAKTYLTLFENVINL---- : 126
PcynK  : QQFGDFMNGIYAYASKKHNIRGAQTEEVKTYLMLFQNVINL---- : 127
PinuiG : KEFGDFINGIYDYSQKKHNIRGTQTEQTKTYHMLFQNVINF---- : 126
PknoE  : QEFKDFMNGIYTYTQKKHNIRGSQTEDIKKYLMLFQNIINYL--- : 127
PcoaE  : QEFKDFMNGIYSYAERKHHIRGAQTEQAKTYLMLFQNVINL---- : 127
PberC  : TEFKEFMNNMYNFVKKNQEGKVMTDNDKM-YMALFENVLSL---- : 122
PyoeC  : TEFKEFMNNMYNFVKKKQEGKEMTENDKI-YMTLFENVLSL---- : 123
PchaC  : AEFKEFMNSMYNFIKKKHEGKTLSENDKQ-YMALFENVLTL---- : 122
PvinvC : DEFKSFMNNMYNFVKKKHEGKLTTETDKQ-YMALFENVLSL---- : 122
PvinpC : AEFKDFMNNMYSFVKKKHEGKTLSESDKQ-YMTLFENVLSL---- : 122
          2Fk F6N 6Y 5  4kh  4       Y  LF2N66 l
```

Comparison 14: Clade 1 Primate-parasites vs Clade 4 Rodent-parasites

```
                    *    +    20    +    +*        40        *  +        60  + +     *         80
PberA  : IKYLDTIYDEILTNSENKNLINDNENHSKYREFKKKYDNFAITPKESEIIKD-LIKMFVTNSNNK--LNELLAVFKKALHDNKFAE :  83
PyoeA  : IKYLDTIYDELLTNSENKNLINNNENHNKYSEFKKRYDNFALTPKESEIIKD-LTKMFVTNSDNK--LNELLVVFKKALHDKEFAE :  83
PchaA  : IKYLDTLYDELLTDDKNQNLID-AENHSKYNEFKKKYDKFAITPKEAEIIKD-LVKMFVTNPDNK--VKELLVVFKKALHDKEFAE :  82
PvinvA : --------------QDSSKGTGSQGHHTKYNELKKKYDHFAITPKEAEIIKDLLVKMFVTNTENK--ANELLAVFKKALTDEEFAE :  70
PvinpA : --YLDTLYDELLTDAKKNSLID-SELHSKYNEFKKKYDKFAITPKEAEIIKDLLVKMFVTNIESK--TNELLAVFKKALHDKEFAE :  81
PberB  : -AFLGTIFDEILNEQNHNEQVHTTQYHSKYNSLKSECD-FAMNLEEYAIAKK-ISSYFKSGTAEN--PIYLYDILIKSLNDEEYKK :  81
PyoeB  : -AFLGAIFDEILKEQHHDDDVHSTEYHSKYNSLKNECD-FEMNLEEYNIAKK-ISSYFQSGSTEN--PIYLYDILLKSLKDEEYKK :  81
PchaB  : -AFLGTVFDEMLKAQDHAKKVHTNEYHSKYNALKGECD-FAMSLEEYEIAKK-ISTYF-NGTTEN--PIHLYDILIKAITDEEYKK :  80
PvinvB : -AFLGTIFDDMLKEQNGNTTINNSEYHSKYNDLKTECD-LSMSTDEYDIAKKLISTYF-SGTTEH--PIKLYDILVKAITDEEYKK :  81
PvinpB : -AFLGTVFDEMLKEQKHDTKIHNTEYHSKYNVLKTECD-FAMSIEEYDIAKKLISTYF-RGTTDN--PIHLYDILIKAITDEEYKK :  81
            l t  de l            H KYn  K   D fa6    E  I K  6   F            L  6  Ka6 D e5

                                     +
              *        100    +     *    + +120     +    *
PberA  : ELNNLISGIYAFSKRHNYLVTEKEEYKEKYEKLYENISKM---- : 123
PyoeA  : ELNNLISGIYAFSKRHNYLVTEKDEYREKYEKLYENISKV---- : 123
PchaA  : EFNNLISGIYAFSKRNNHLVIDQKEYKDKYNKLYEHISNL---- : 122
PvinvA : EFDNIISGIYAFSKKNNHLVIDQVEYKEKYGKLYETMSKL---- : 110
PvinpA : EFNNLISGIYAFSKKYNHLVIDQVEYKEKYDKLYE--------- : 116
PberB  : HFKNFIYGVYSFAKKYNYLSESRLE-EEN--------------- : 109
PyoeB  : HFKNFIYGIYSFAKKYNYLSATRLA-EANSQFIPNVLK------ : 118
PchaB  : HFKNFIYGIYSFAKKHNYLSTARLA-EENSQFISNVLNVL---- : 119
PvinvB : HFKNFIYVIYSFAKKYNYLSKTRLE-DENKQFITNVLN------ : 118
PvinpB : HFKNFIYGIYSFAKKYNYLSKTRLA-EENSQFIRNVLN------ : 118
          f N I g6Y F K4 N L        e
```

Comparison 16: Clade 3d Primate-parasites (B/E) vs Clade 2 Primate-parasites

```
                  *        20      +  *        40  +      *     +  60         *   +    80
PvivB  : IKYLDKLYDEVLTTSDNT--SGIHVPDYHSKYNTIRQKYE--YSMNPVEYEIVKN-FNVGFKNDGAASSDATPLVDVFKKALADEK :  81
PcynB  : IKYMDKLYDDILSTLGKT--NEIHIPLYHSKYNTIRKDYE--LSMKPVEYQIVKNLFNVGFKKEGE-SSAANSLTEVFKKVLVDEK :  81
PvivE  : IKYLDKLYDEVLTTSDNT--NGIHVPDYHSKYNTIRQKYE--YSMNPVEYEIVKN-FNVGFKNDGAASSDATPLVDVFKKALADET :  81
PcynE  : IKYMDKLYDDILSTSGKT--NEIHIPLYHSKYNTIRKDYE--LSMKPVEYQIVKNLFNVGFKKEGE-SSAANSLTEVFKKVLVDEK :  81
PvivK  : LKYSDELYEDILTSLNKK--GCEEGTENNNKYNEFKKEYEMFISLNKEEYEIIEK-VDAFSMYNDSIDEDADSVYEAIKKSFTDPK :  83
PcynK  : LKYSDELYEDILMNLHKK--GGKEGTDYNDKYNEFKKEYDMFISLNKDEYEIISKLVNAFSMYNDAIDEDADTVYEAIKKSFTDPK :  84
PinuiG : -KYSEKVYEDILISLNKR--GGEQGTNENHKYNEFKKEYDMFISLNKDEYEIIEKLVDAFCVNNQPFDEDADSVYEAIKKSFTDPK :  83
PknoE  : LNYADDLYEDILSSLSKK--GGEEGTNYDDKYNEFKKEYDRFISLSKDEYEIIGK-IDAFSMYNDAISEDTDSVYQAIKKSFTDKK :  83
PcoaE  : LDYADELYEEILSSLSKK--DGEEGTNYDDKYNDFKKEYDMFISLSKDEYEIIGKLIDAFSMYNDGINEDADSVYEAIKKSFTDPK :  84
          kY d 6Y  6L    k          y  KYN  4k Y    S6   EY2I6    1           da  6    KK   D k

                                        +
            *      100          *      120         *
PvivB  : FQAEFDNFVHGLYGFAKRHSYLSEARMKDNKLYSDLLKNAISL- : 124
PcynB  : FQDEFNNFVHGLYGFAKRHNYLGKERLENTTSDSDLLKNAFSL- : 124
PvivE  : FQAEFDNFVHGLYGFAKRHNYLSEARMKDADRYTNLLKNAISL- : 124
PcynE  : FQDEFNNFVHGLYGFAKRHNYLGNKRLENTTVDSDLLKNAFSL- : 124
PvivK  : FRKEFKDFMNGIYAYAKRKHYIRGTQTEKAKTYLTLFENVINL- : 126
PcynK  : FKQQFGDFMNGIYAYASKKHNIRGAQTEEVKTYLMLFQNVINL- : 127
PinuiG : FKKEFGDFINGIYDYSQKKHNIRGTQTEQTKTYHMLFQNVINF- : 126
PknoE  : FKQEFKDFMNGIYTYTQKKHNIRGSQTEDIKKYLMLFQNIINYL : 127
PcoaE  : FKQEFKDFMNGIYSYAERKHHIRGAQTEQAKTYLMLFQNVINL- : 127
         F  2F 1F6 G6Y 5a 4   6     e    y  L  N i l
```

Comparison 18: Clade 3d Primate-parasites (B/E) vs Clade 4 Rodent-parasites

```
                                               +
                  *        20    +    *        40         *        60         *+       80
PvivB  : IKYLDKLYDEVLTTSDNTSGIHVPDYHSKYNTIRQKYEYSMNPVEYEIVKN-FNVGFKNDGAASSDATPLVDVFKKALADEKFQAE :  85
PcynB  : IKYMDKLYDDILSTLGKTNEIHIPLYHSKYNTIRKDYELSMKPVEYQIVKNLFNVGFKKEG-ESSAANSLTEVFKKVLVDEKFQDE :  85
PvivE  : IKYLDKLYDEVLTTSDNTNGIHVPDYHSKYNTIRQKYEYSMNPVEYEIVKN-FNVGFKNDGAASSDATPLVDVFKKALADETFQAE :  85
PcynE  : IKYMDKLYDDILSTSGKTNEIHIPLYHSKYNTIRKDYELSMKPVEYQIVKNLFNVGFKKEG-ESSAANSLTEVFKKVLVDEKFQDE :  85
PberB  : -AFLGTIFDEILNEQNHNEQVHTTQYHSKYNSLKSECDFAMNLEEYAIAKK-ISSYFKS-G-TAENPIYLYDILIKSLNDEEYKKH :  82
PyoeB  : -AFLGAIFDEILKEQHHDDDVHSTEYHSKYNSLKNECDFPMNLEEYNIAKK-ISSYFQS-G-STENPIYLYDILLKSLKDEEYKKH :  82
PchaB  : -AFLGTVFDEMLKAQDHAKKVHTNEYHSKYNALKGECDFAMSLEEYEIAKK-ISTYFN--G-TTENPIHLYDILIKAITDEEYKKH :  81
PvinvB : -AFLGTIFDDMLKEQNGNTTINNSEYHSKYNDLKTECDLSMSTDEYDIAKKLISTYFS--G-TTEHPIKLYDILVKAITDEEYKKH :  82
PvinpB : -AFLGTVFDEMLKEQKHDTKIHNTEYHSKYNVLKTECDFAMSIEEYDIAKKLISTYFR--G-TTDNPIHLYDILIKAITDEEYKKH :  82
           56  65D 6L        6h   YHSKYN 64      M   EY I K      F   G        L d6  K 6 DE 5

                                          +
            *       100         *   +  +120    +
PvivB  : FDNFVHGLYGFAKRHSYLSEARMKDNKLYSDLLKNAISL- : 124
PcynB  : FNNFVHGLYGFAKRHNYLGKERLENTTSDSDLLKNAFSL- : 124
PvivE  : FDNFVHGLYGFAKRHNYLSEARMKDADRYTNLLKNAISL- : 124
PcynE  : FNNFVHGLYGFAKRHNYLGNKRLENTTVDSDLLKNAFSL- : 124
PberB  : FKNFIYGVYSFAKKYNYLSESRLEEEN------------- : 109
PyoeB  : FKNFIYGIYSFAKKYNYLSATRLAEAN--SQFIPNVLK-- : 118
PchaB  : FKNFIYGIYSFAKKHNYLSTARLAEEN--SQFISNVLNVL : 119
PvinvB : FKNFIYVIYSFAKKYNYLSKTRLEDEN--KQFITNVLN-- : 118
PvinpB : FKNFIYGIYSFAKKYNYLSKTRLAEEN--SQFIRNVLN-- : 118
         F NF6 g6Y FAK4 nYLs  R6           n
```

Comparison 20: Clade 2 Primate-parasites vs Clade 3d Primate-parasites (C/H/E/D/I)

```
                                                                               +
                   *        20         *        40         *   +  + 60    + +  *+  +    80   +
PvivK   : LKYSDELYEDILTSLNKKGCEEGTENNNKYNEFKKEYEMFISLNKEEYEIIEK-VDAFSMYNDSIDEDADSVYEAIKKSFTDPK :  83
PcynK   : LKYSDELYEDILMNLHKKGGKEGTDYNDKYNEFKKEYDMFISLNKDEYEIISKLVNAFSMYNDAIDEDADTVYEAIKKSFTDPK :  84
PinuiG  : -KYSEKVYEDILISLNKRGGEQGTNENHKYNEFKKEYDMFISLNKDEYEIIEKLVDAFCVNNQPFDEDADSVYEAIKKSFTDPK :  83
PknoE   : LNYADDLYEDILSSLSKKGGEEGTNYDDKYNEFKKEYDRFISLSKDEYEIIGK-IDAFSMYNDAISEDTDSVYQAIKKSFTDKK :  83
PcoaE   : LDYADELYEEILSSLSKKDGEEGTNYDDKYNDFKKEYDMFISLSKDEYEIIGKLIDAFSMYNDGINEDADSVYEAIKKSFTDPK :  84
PvivC   : VKYLDKLYDDVLATQDSANGIDVPPFHSKYNDFRKKYEF--KMNDSEYKIMKNFDVSF--KKEGQQSSAACLVNVFKKVLDDEH :  80
PcynC   : VKYLDKLYDEVLETPNSVNAIHTQPFHSKYNEFRKKYEF--TMNEREYQIVKNLFDVCF-KKEGEQSSTSCIVNMFKKVLDDKN :  81
PvivH   : VKYLDKLYDEVLKTTDAKDEIHVPPFHSKYNDFRKKYEF--TMNEREYQIVKN-FDAF--FKKDGNPSPADAVSFFKKMLNDPN :  79
PcynH   : VKYLDKLYDEVLNTSEEKNAIHVPAFHSKYNDFRTKYEF--TMNEQEYHIVKKLFDAF--FKKEQNSSYDAPIKFFKNVLNDAE :  80
PknoD   : VKYLDKLYDEVLKGEDGKNGIHIPEFHSKYNDFRKKYEL--TMNEQEYQMMKK-FDAF--FKKGESTNAVCPLEFFKKVLNNMS :  79
PcoaD   : VKYLDKLYDEVLNTENANNGIHVHPYHSKYNDFRKNYEF--TMNEQEYQIMKKLFDVF--FKKEEGSNSVCPIAFFKKVLDDLS :  80
PinuiE  : VKYLDKLYDELLKTTEGKDGIHVPLFHSKYNDFRKKYEH--TMNEREYQIVKNLFEGF--F-KEGHSKCGCPVEFFKKLLNDAG :  79
PvivI   : VKYLDKLYDEVLKTTDAKDEIHVPPFHSKYNDFRKKYEF--TMNEREYQIVKN-FDAF--FKKDGNPSPADAVSFFKKMLNDPN :  79
PcynI   : VKYLDKLYDEILKTTDVKDGIHVPAFHSKYNDFRTKYEF--TMNEREYQIVKNLFDTF--FKKDGNSNDDAPIKFFKNVLNDAD :  80
           kY d 6Y  6L                KYN F4k Y     6n  EY 66      f                  Kk   1

                                          +
             + *  ++   100         *  ++   120  + +
PvivK   : FRKEFKDFMNGIYAYAKRKHYIRGTQTEKAKTYLTLFENVINL- : 126
PcynK   : FKQQFGDFMNGIYAYASKKHNIRGAQTEEVKTYLMLFQNVINL- : 127
PinuiG  : FKKEFGDFINGIYDYSQKKHNIRGTQTEQTKTYHMLFQNVINF- : 126
PknoE   : FKQEFKDFMNGIYTYTQKKHNIRGSQTEDIKKYLMLFQNIINYL : 127
PcoaE   : FKQEFKDFMNGIYSYAERKHHIRGAQTEQAKTYLMLFQNVINL- : 127
PvivC   : LQKEFDNFVHGFYGFAKRHNYLRGERMANENLYKDIFKNVVNL- : 123
PcynC   : FQKKFDNFVQGFYGFAKRHNYLRGERMANENLYKDILKNVVNL- : 124
PvivH   : VQKEFDNFVHGLYGFAKRHNYLRGERMTDTKLYDELLKNVVNL- : 122
PcynH   : FQKQFDNFQHGLYGFAKRHNYLRGDRMTDGQLYSELLKNVINL- : 123
PknoD   : LQEEFDNFQHGLYGFAKRHNYLRGEKTTNEKLYHDLLKNIINL- : 122
PcoaD   : LQKQFDNFQKGLYGFTKRHNYLRGEKTTNGQLYDELIKNIINL- : 123
PinuiE  : LQKQFDNFMHGLYGFAKRHNYLRGERMTDRKLCEEVLKNVINL- : 122
PvivI   : VQKEFDNFVHGLYGFAKRHNYLRDERMNLTDAHKSLFVNTLSL- : 122
PcynI   : FQKQFDNFQHGLYGFAKRHNYLRGQRMNLTDAHKNLFLNALSL- : 123
              F 1F  G Y 5a 4   6Rg        y  6  N 6nl
```

Comparison 21: Clade 3d Primate-parasites (C/H/E/D/I) vs Clade 2 Rodent-parasites

```
                                                                              +
                  *        20         *        40         *   +  +  60    + +  *+  +    80   +
PvivC  : VKYLDKLYDDVLATQDSANGIDVPPFHSKYNDFRKKYEF--KMNDSEYKIMKNFDVSF--KKEGQQSSAACLVNVFKKVLDDEH :  80
PcynC  : VKYLDKLYDEVLETPNSVNAIHTQPFHSKYNEFRKKYEF--TMNEREYQIVKNLFDVCF-KKEGEQSSTSCIVNMFKKVLDDKN :  81
PcynH  : VKYLDKLYDEVLNTSEEKNAIHVPAFHSKYNDFRTKYEF--TMNEQEYHIVKKLFDAF--FKKEQNSSYDAPIKFFKNVLNDAE :  80
PvivH  : VKYLDKLYDEVLKTTDAKDEIHVPPFHSKYNDFRKKYEF--TMNEREYQIVKN-FDAF--FKKDGNPSPADAVSFFKKMLNDPN :  79
PknoD  : VKYLDKLYDEVLKGEDGKNGIHIPEFHSKYNDFRKKYEL--TMNEQEYQMMKK-FDAF--FKKGESTNAVCPLEFFKKVLNNMS :  79
PcoaD  : VKYLDKLYDEVLNTENANNGIHVHPYHSKYNDFRKNYEF--TMNEQEYQIMKKLFDVF--FKKEEGSNSVCPIAFFKKVLDDLS :  80
PinuiE : VKYLDKLYDELLKTTEGKDGIHVPLFHSKYNDFRKKYEH--TMNEREYQIVKNLFEGF--F-KEGHSKCGCPVEFFKKLLNDAG :  79
PvivI  : VKYLDKLYDEVLKTTDAKDEIHVPPFHSKYNDFRKKYEF--TMNEREYQIVKN-FDAF--FKKDGNPSPADAVSFFKKMLNDPN :  79
PcynI  : VKYLDKLYDEILKTTDVKDGIHVPAFHSKYNDFRTKYEF--TMNEREYQIVKNLFDTF--FKKDGNSNDDAPIKFFKNVLNDAD :  80
PberC  : -NYTNDFYNAVIASLNENDKNGN--YKNKYEDFKNKYDNVIAINEKEFEIFSK-VEVFMKNHHDFNLNSNFLYELLTKALSDQN :  80
PyoeC  : -NYTNDFYNAVIANLNENDKNGN--YKNKYEDFKNKYDNVIAIDEKEFEIFSKLVEVFMKNHQDFSFNSNFLYELLTKALSDQG :  81
PchaC  : QNYTDDFFNSVIANINENDNGD---YKDKYEDFKSKYDNIIAINQKEFEIFSK-IEGFMQSNKDMNLSSDYLYEVLTKALSDKA :  80
PvinvC : -NYADDIFGSVIANINTSDNGD---YKVKYEDFKNKYDNLIAIDQTEFEIFSKLVEGFMKTNKEINLSSDYLYDVLTKTLSDKA :  80
PvinpC : -NYTDDFFNSVIANINTSDNGE---YKIKYEDFKNKYDNIMAIDQREFEIFSKLVEGFMKNNKEMNLSSDYLYEVITKALSDTA :  80
           Y 1  5  66      1      5  KY dF4 kY     61  E5 6       f                   k L 1

                                          +
            + *  ++   100         * ++    120  + +
PvivC  : LQKEFDNFVHGFYGFAKRHNYLRGERMANENLYKDIFKNVVNL- : 123
PcynC  : FQKKFDNFVQGFYGFAKRHNYLRGERMANENLYKDILKNVVNL- : 124
PcynH  : FQKQFDNFQHGLYGFAKRHNYLRGDRMTDGQLYSELLKNVINL- : 123
PvivH  : VQKEFDNFVHGLYGFAKRHNYLRGERMTDTKLYDELLKNVVNL- : 122
PknoD  : LQEEFDNFQHGLYGFAKRHNYLRGEKTTNEKLYHDLLKNIINL- : 122
PcoaD  : LQKQFDNFQKGLYGFTKRHNYLRGEKTTNGQLYDELIKNIINL- : 123
PinuiE : LQKQFDNFMHGLYGFAKRHNYLRGERMTDRKLCEEVLKNVINL- : 122
PvivI  : VQKEFDNFVHGLYGFAKRHNYLRDERMNLTDAHKSLFVNTLSL- : 122
PcynI  : FQKQFDNFQHGLYGFAKRHNYLRGQRMNLTDAHKNLFLNALSL- : 123
PberC  : FKTEFKEFMNNMYNFVKKNQEGK-VMTDNDKMYMALFENVLSL- : 122
PyoeC  : FKTEFKEFMNNMYNFVKKKQEGK-PMTENDKIYMTLFENVLSL- : 123
PchaC  : FKAEFKEFMNSMYNFIKKKHEGK-TLSENDKQYMALFENVLTL- : 122
PvinvC : FKDEFKSFMNNMYNFVKKKHEGK-LTTETDKQYMALFENVLSL- : 122
PvinpC : FKAEFKDFMNNMYSFVKKKHEGK-TLSESDKQYMTLFENVLSL- : 122
             F  F    Y F K4    4         y  6  N 6 L
```

Comparison 22: Clade 3d Primate-parasites (C/H/E/D/I) vs Clade 4 Rodent-parasites

```
                      *       20         *       40         *   +    60+  +  ++ *+        80+
PvivC  : VKYLDKLYDDVLATQDSANGIDVPPFHSKYNDFRKKYEFKMNDSEYKIMKN-FDVSFKKEGQQSSAACLVNVFKKVLDDEHLQK :  83
PcynC  : VKYLDKLYDEVLETPNSVNAIHTQPFHSKYNEFRKKYEFTMNEREYQIVKNLFDVCFKKEGEQSSTSCIVNMFKKVLDDKNFQK :  84
PvivH  : VKYLDKLYDEVLKTTDAKDEIHVPPFHSKYNDFRKKYEFTMNEREYQIVKN-FDAFFKKDG-NPSPADAVSFFKKMLNDPNVQK :  82
PcynH  : VKYLDKLYDEVLNTSEEKNAIHVPAFHSKYNDFRTKYEFTMNEQEYHIVKKLFDAFFKKEQ-NSSYDAPIKFFKNVLNDAEFQK :  83
PknoD  : VKYLDKLYDEVLKGEDGKNGIHIPEFHSKYNDFRKKYELTMNEQEYQMMKK-FDAFFKKGE-STNAVCPLEFFKKVLNNMSLQE :  82
PcoaD  : VKYLDKLYDEVLNTENANNGIHVHPYHSKYNDFRKNYEFTMNEQEYQIMKKLFDVFFKKEE-GSNSVCPIAFFKKVLDDLSLQK :  83
PinuiE : VKYLDKLYDELLKTEGKDGIHVPLFHSKYNDFRKKYEHTMNEREYQIVKNLFEGFF-KEG-HSKCGCPVEFFKKLLNDAGLQK :  82
PvivI  : VKYLDKLYDEVLKTTDAKDEIHVPPFHSKYNDFRKKYEFTMNEREYQIVKN-FDAFFKKDG-NPSPADAVSFFKKMLNDPNVQK :  82
PcynI  : VKYLDKLYDEILKTTDVKDGIHVPAFHSKYNDFRTKYEFTMNEREYQIVKNLFDTFFKKDG-NSNDDAPIKFFKNVLNDADFQK :  83
PberB  : -AFLGTIFDEILNEQNHNEQVHTTQYHSKYNSLKSECDFAMNLEEYAIAKK-ISSYFKSGT-AENPIYLYDILIKSLNDEEYKK :  81
PyoeB  : -AFLGAIFDEILKEQHHDDDVHSTEYHSKYNSLKNECDFPMNLEEYNIAKK-ISSYFQSGS-TENPIYLYDILLKSLKDEEYKK :  81
PchaB  : -AFLGTVFDEMLKAQDHAKKVHTNEYHSKYNALKGECDFAMSLEEYEIAKK-ISTYF-NGT-TENPIHLYDILIKAITDEEYKK :  80
PvinvB : -AFLGTIFDDMLKEQNGNTTNNSEYHSKYNDLKTECDLSMSTDEYDIAKKLISTYF-SGT-TEHPIKLYDILVKAITDEEYKK :  81
PvinpB : -AFLGTVFDEMLKEQKHDTKIHNTEYHSKYNVLKTECDFAMSIEEYDIAKKLISTYF-EGT-TDNPIHLYDILIKAITDEEYKK :  81
            5L  65De6L        6h    5HSKYN  4     f Mn   EY 6 K       F                    k 6 1     k

             ++                  ++      +
         +     *         100       *     +  120+  +
PvivC  : EFDNFVHGFYGFAKRHNYLRGERMANENLYKDIFKNVVNL- : 123
PcynC  : KFDNFVQGFYGFAKRHNYLRGERMANENLYKDILKNVVNL- : 124
PvivH  : EFDNFVHGLYGFAKRHNYLRGERMTDTKLYDELLKNVVNL- : 122
PcynH  : QFDNFQHGLYGFAKRHNYLRGDRMTDGQLYSELLKNVINL- : 123
PknoD  : EFDNFQHGLYGFAKRHNYLRGEKTTNEKLYHDLLKNIINL- : 122
PcoaD  : QFDNFQKGLYGFTKRHNYLRGEKTTNGQLYDELIKNIINL- : 123
PinuiE : QFDNFMHGLYGFAKRHNYLRGERMTDRKLCEEVLKNVINL- : 122
PvivI  : EFDNFVHGLYGFAKRHNYLRDERMNLTDAHKSLFVNTLSL- : 122
PcynI  : QFDNFQHGLYGFAKRHNYLRGQRMNLTDAHKNLFLNALSL- : 123
PberB  : HFKNFIYGVYSFAKKYNYLSESRLEEEN------------- : 109
PyoeB  : HFKNFIYGIYSFAKKYNYLSATRLAEAN--SQFIPNVLK-- : 118
PchaB  : HFKNFIYGIYSFAKKHNYLSTARLAEEN--SQFISNVLNVL : 119
PvinvB : HFKNFIYVIYSFAKKYNYLSKTRLEDEN--KQFITNVLN-- : 118
PvinpB : HFKNFIYGIYSFAKKYNYLSKTRLAEEN--SQFIRNVLN-- : 118
          F NF  g Y FaK4 NYL   4            n
```

Comparison 23: Clade 2 Primate-parasites vs Clade 4 Rodent-parasites

```
                  *         20         *         40         *   +     60         *         80
PvivK  : LKYSDELYEDILTSLNKKGCEEGTENNNKYNEFKKEYEMFISLNKEEYEIIEK-VDAFSMYNDSIDEDADSVYEAIKKSFTDPKFR :  85
PcynK  : LKYSDELYEDILMNLHKKGGKEGTDYNDKYNEFKKEYDMFISLNKDEYEIISKLVNAFSMYNDAIDEDADTVYEAIKKSFTDPKFK :  86
PinuiG : -KYSEKVYEDILISLNKRGGEQGTNENHKYNEFKKEYDMFISLNKDEYEIIEKLVDAFCVNNQPFDEDADSVYEAIKKSFTDPKFK :  85
PknoE  : LNYADDLYEDILSSLSKKGGEEGTNYDDKYNEFKKEYDRFISLSKDEYEIIGK-IDAFSMYNDAISEDTDSVYQAIKKSFTDKKFK :  85
PcoaE  : LDYADELYEEILSSLSKKDGEEGTNYDDKYNDFKKEYDMFISLSKDEYEIIGKLIDAFSMYNDGINEDADSVYEAIKKSFTDPKFK :  86
PyoeB  : -AFLGAIFDEILKEQHHDDDVHSTEYHSKYNSLKNECD--FPMNLEEYNIAKK-ISSYFQ--SGSTENPIYLYDILLKSLKDEEYK :  80
PberB  : -AFLGTIFDEILNEQNHNEQVHTTQYHSKYNSLKSECD--FAMNLEEYAIAKK-ISSYFK--SGTAENPIYLYDILIKSLNDEEYK :  80
PvinvB : -AFLGTIFDDMLKEQNGNTTINNSEYHSKYNDLKTECD--LSMSTDEYDIAKKLISTYF---SGTTEHPIKLYDILVKAITDEEYK :  80
PvinpB : -AFLGTVFDEMLKEQKHDTKIHNTEYHSKYNVLKTECD--FAMSIEEYDIAKKLISTYF---RGTTDNPIHLYDILIKAITDEEYK :  80
PchaB  : -AFLGTVFDEMLKAQDHAKKVHTNEYHSKYNALKGECD--FAMSLEEYEIAKK-ISTYF---NGTTENPIHLYDILIKAITDEEYK :  79
           5   65  6L             y  KYN  K E d    6   EY I  K 6  5        e    6Y  6 K  tD  54

                                         +
            *       100         *       120 +    +
PvivK  : KEFKDFMNGIYAYAKRKHYIRGTQTEKAKTYLTLFENVINL- : 126
PcynK  : QQFGDFMNGIYAYASKKHNIRGAQTEEVKTYLMLFQNVINL- : 127
PinuiG : KEFGDFINGIYDYSQKKHNIRGTQTEQTKTYHMLFQNVINF- : 126
PknoE  : QEFKDFMNGIYTYTQKKHNIRGSQTEDIKKYLMLFQNIINYL : 127
PcoaE  : QEFKDFMNGIYSYAERKHHIRGAQTEQAKTYLMLFQNVINL- : 127
PyoeB  : KHFKNFIYGIYSFAKKYNYLSATRLAEANS--QFIPNVLK-- : 118
PberB  : KHFKNFIYGVYSFAKKYNYLSESRLEEEN------------- : 109
PvinvB : KHFKNFIYVIYSFAKKYNYLSKTRLEDENK--QFITNVLN-- : 118
PvinpB : KHFKNFIYGIYSFAKKYNYLSKTRLAEENS--QFIRNVLN-- : 118
PchaB  : KHFKNFIYGIYSFAKKHNYLSTARLAEENS--QFISNVLNVL : 119
           Fk1F6 g6Y 5a 4   6                n  n
```

Comparison 24: Clade 3c Primate-parasites (G/C/D) vs Clade 3d Primate-parasites (C/H/E/D/I)

```
                                            +
                  *         +20  +    +  *         40          *    +   +60+ + +  +  +*        80 +
PvivG  : IKYLDKLYDEVLTTSDNTSGIHVPDYHSKYNTIRQKYEYSMNPVEYEIVKN-FNVGFKNDGAASSDATPLVDVFKKALADEKFQ :  83
PcynG  : IKYMDKLYDDILSTSGNTNEIHIPLYHSKYNTIRKDYELSMKPVEYQIVKNLFNVGFKKEGESSAA-NSLTEVFKKVLVDEKFQ :  83
PinuiD : IKYLDKLYDEIMNISDRTNDINKQAYDSKYSTIKKKYKLSMNPVEYQIVKNLFNVGFKKEGETSVT-TSLSEVFKKVLDDEKSQ :  83
PknoC  : INYLDKLYDEIITTPDIKKKMQNSSY-------KTKYKLSLNPAEYQIVKK-FELGFGNENESSAG-TSLVEVFKKVLDDEKFQ :  75
PcoaC  : ITFLDKLYDDIINTSDSKNEIHNTPYHTKYNAIRNKYELSMNPVEYQIVKNLFEVGFNKEGESSAD-TSFIDIFKKVLDDEKFQ :  83
PvivC  : VKYLDKLYDDVLATQDSANGIDVPPFHSKYNDFRKKYEFKMNDSEYKIMKN-FDVSFKKEGQQSSA-ACLVNVFKKVLDDEHLQ :  82
PcynC  : VKYLDKLYDEVLETPNSVNAIHTQPFHSKYNEFRKKYEFTMNEREYQIVKNLFDVCFKKEGEQSST-SCIVNMFKKVLDDKNFQ :  83
PvivH  : VKYLDKLYDEVLKTTDAKDEIHVPPFHSKYNDFRKKYEFTMNEREYQIVKN-FDAFFKKDGNPSPA--DAVSFFKKMLNDPNVQ :  81
PcynH  : VKYLDKLYDEVLNTSEEKNAIHVPAFHSKYNDFRTKYEFTMNEQEYHIVKKLFDAFFKKEQNSSYD--APIKFFKNVLNDAEFQ :  82
PcoaD  : VKYLDKLYDEVLNTENANNGIHVHPYHSKYNDFRKNYEFTMNEQEYQIMKKLFDVFFKKEEGSNSV--CPIAFFKKVLDDLSLQ :  82
PknoD  : VKYLDKLYDEVLKGEDGKNGIHIPEFHSKYNDFRKKYELTMNEQEYQMMKK-FDAFFKK-GESTNA-VCPLEFFKKVLNNMSLQ :  81
PinuiE : VKYLDKLYDELLKTTEGKDGIHVPLFHSKYNDFRKKYEHTMNEREYQIVKNLFEGFF-KEGHSKCG--CPVEFFKKLLNDAGLQ :  81
PvivI  : VKYLDKLYDEVLKTTDAKDEIHVPPFHSKYNDFRKKYEFTMNEREYQIVKN-FDAFFKKDGNPSPA--DAVSFFKKMLNDPNVQ :  81
PcynI  : VKYLDKLYDEILKTTDVKDGIHVPAFHSKYNDFRTKYEFTMNEREYQIVKNLFDTFFKKDGNSNDD--APIKFFKNVLNDADFQ :  82
         6k56DkLYDe66 t      6h   5h kyn  4 kYe  6n  EY 66K  F   Fkk g             FKk L l    Q

                                   +
          +    *++      100       *++     +120 +
PvivG  : AEFDNFVHGLYGFAKRHSYLSKERMGDTTRYSDLLKNAISL : 124
PcynG  : DEFNNFVHGLYGFAKRHNYLGKERLENTTSDSDLLKNAFSL : 124
PinuiD : EQFDNFVEGLYGFAKRHSYLGKDGMDN-TRYSDLLKNAISL : 123
PknoC  : KEFDNIVQGFYGFAKRHNYISQEQMNN-TSHTNLLKNAINL : 115
PcoaC  : DEFDNFVQGLYGFAKRHNYLGKERMDNTTSYSNLLKNALSL : 124
PvivC  : KEFDNFVHGFYGFAKRHNYLRGERMANENLYKDIFKNVVNL : 123
PcynC  : KKFDNFVQGFYGFAKRHNYLRGERMANENLYKDILKNVVNL : 124
PvivH  : KEFDNFVHGLYGFAKRHNYLRGERMTDTKLYDELLKNVVNL : 122
PcynH  : KQFDNFQHGLYGFAKRHNYLRGDRMTDGQLYSELIKNVINL : 123
PcoaD  : KQFDNFQKGLYGFTKRHNYLRGEKTTNGQLYDELIKNIINL : 123
PknoD  : EEFDNFQHGLYGFAKRHNYLRGEKTTNEKLYHDLIKNIINL : 122
PinuiE : KQFDNFMHGLYGFAKRHNYLRGERMTDRRLCEEVLKNVINL : 122
PvivI  : KEFDNFVHGLYGFAKRHNYLRDERMNLTDAHKSLFVNTISL : 122
PcynI  : KQFDNFQHGLYGFAKRHNYLRGQRMNLTLAHKNLFLNALSL : 123
           F1Nf  GlYGFaKRHnY6            6 kN    L
```

Comparison 25: Clade 2 Primate-parasites vs Clade 3c Primate-parasites (G/C/D)

```
                                          +                                          +
                       *       +20       +  *        40         *   + +  60         *        80
PvivK  : LKYSDELYEDILTSLNKKGCEEGTENNNKYNEFKKEYEMFISLNKEEYEIIEK-VDAFSMYNDSIDEDADSVYEAIKKSFTDPKFR :  85
PcynK  : LKYSDELYEDILMNLHKKGGKEGTDNNDKYNEFKKEYDMFISLNKDEYEIISKLVNAFSMYNDAIDEDADTVYEAIKKSFTDPKFK :  86
PinuiG : -KYSEKVYEDILISLNKRGGEQGTNENHKYNEFKKEYDMFISLNKDEYEIIEKLVDAFCVNNQPFDEDADSVYEAIKKSFTDPKFK :  85
PknoE  : LNYADDLYEDILSSLSKKGGEEGTNYDDKYNEFKKEYDRFISLSKDEYEIIGK-IDAFSMYNDAISEDTDSVYQAIKKSFTDKKFK :  85
PcoaE  : LDYADELYEEILSSLSKKDGEEGTNYDDKYNDFKKEYDMFISLSKDEYEIIGKLIDAFSMYNDGINEDADSVYEAIKKSFTDPKFK :  86
PvivG  : IKYLDKLYDEVLTTSDNTSGIHVPDYHSKYNTIRQKYE--YSMNPVEYEIVKN-FNVGFKNDGAASSDATPLVDVFKKALADEKFQ :  83
PcynG  : IKYMDKLYDDILSTSGNTNEIHIPLYHSKYNTIRKDYE--LSMKPVEYQIVKNLFNVGFKKEGESSAA-NSLTEVFKKVLVDEKFQ :  83
PinuiD : IKYLDELYDEIMNISDRTNDINKQAYDSKYSTIKKKYK--LSMNPVEYQIVKNLFNVGFKKEGETSVT-TSLSEVFKKVLDDEKSQ :  83
PknoC  : INYLDELYDEIITTPDIKKKMQNSSYKTKYK---------LSLNPAEYQIVKK-FELGFGNENESSAG-TSLVEVFKKVLDDEKFQ :  75
PcoaC  : ITFLDKLYDDIINTSDSKNEIHNTPYHTKYNAIRNKYE--LSMNPVEYQIVKNLFEVGFNKEGESSADTSFI-DIFKKVLDDEKFQ :  83
           5 d 6Y  66               y  KYn     y    S6   EY2I6                    6    KK   D Kf

          *        100        *       120
PvivK  : KEFKDFMNGIYAYAKRKHHYIRGTQTEKAKTYLTLFENVINL- : 126
PcynK  : QQFGDFMNGIYAYASKKHHNIRGAQTEEVKTYLMLFQNVINL- : 127
PinuiG : KEFGDFINGIYDYSQKKHHNIRGTQTEQTKTYHMLFQNVINF- : 126
PknoE  : QEFKDFMNGIYTYTQKKHHNIRGSQTEDIKKYLMLFQNIINYL : 127
PcoaE  : QEFKDFMNGIYSYAERKHHHIRGAQTEQAKTYLMLFQNVINL- : 127
PvivG  : AEFDNFVHGLYGFAKRHSYLSKERMGDTTRYSDLLKNAISL- : 124
PcynG  : DEFNNFVHGLYGFAKRHNYLGKERLENTTSDSDLLKNAFSL- : 124
PinuiD : EQFDNFVEGLYGFAKRHSYLGKDGMENTR-YSDLLKNAISL- : 123
PknoC  : KEFDNIVQGFYGFAKRHNYISQEQMNNTS-HTNLLKNAINL- : 115
PcoaC  : DEFDNFVQGLYGFAKRHNYLGKERMDNTTSYSNLLKNALSL- : 124
           2F 1f6 G Y 5a 4   6         y  L  N   l
```

Comparison 27: Clade 3c Primate-parasites (G/C/D) vs Clade 4 Rodent-parasites

```
                                         +
                  *        +20        + *         40          *   +   +60         *          80
PvivG  : IKYLDKLYDEVLTTSDNTSGIHVPDYHSKYNTIRQKYEYSMNPVEYEIVKN-FNVGFKNDGAASSDATPLVDVFKKALADEKFQAE :  85
PcynG  : IKYMDKLYDDILSTSGNTNEIHIPLYHSKYNTIRKDYELSMKPVEYQIVKNLFNVGFKKEG-ESSAANSLTEVFKKVLVDEKFQLE :  85
PinuiD : IKYLDELYDEIMNISDRTNDINKQAYDSKYSTIKKKYKLSMNPVEYQIVKNLFNVGFKKEG-ETSVTTSLSEVFKKVLDDEKSQEQ :  85
PknoC  : INYLDELYDEIITTPDIKKKMQNSSYKTKY-------KLSLNPAEYQIVKK-FELGFGNEN-ESSAGTSLVEVFKKVLDDEKFQKE :  77
PcoaC  : ITFLDKLYDDIINTSDSKNEIHNTPYHTKYNAIRNKYELSMNPVEYQIVKNLFEVGFNKEG-ESSADTSFIDIFKKVLDDEKFQDE :  85
PberB  : -AFLGTIFDEILNEQNHNEQVHTTQYHSKYNSLKSECDFAMNLEEYAIAKK-ISSYFKS-G-TAENPIYLYDILIKSLNDEEYKKH :  82
PyoeB  : -AFLGAIFDEILKEQHHDDDVHSTEYHSKYNSLKNECDFPMNLEEYNIAKK-ISSYFQS-G-STENPIYLYDILLKSLKDEEYKKH :  82
PchaB  : -AFLGTVFDEMLKAQDHAKKVHTNEYHSKYNALKGECDFAMSLEEYEIAKK-ISTYFN--G-TTENPIHLYDILIKAITDEEYKKH :  81
PvinvB : -AFLGTIFDDMLKEQNGNTTINNSEYHSKYNDLKTECDLSMSTDEYDIAKKLISTYFS--G-TTEHPIKLYDILVKAITDEEYKKH :  82
PvinpB : -AFLGTVFDEMLKEQKHDTKIHNTEYHSKYNVLKTECDFAMSIEEYDIAKKLISTYFR--G-TTDNPIHLYDILIKAITDEEYKKH :  82
            56   65D 66        6    Yh3KYn          6    EY I K      F   g        l  6   K 6 DE

             *        100        *       120
PvivG  : FDNFVHGLYGFAKRHSYLSKERMGDTTRYSDLLKNAISL- : 124
PcynG  : FNNFVHGLYGFAKRHNYLGKERLENTTSDSDLLKNAFSL- : 124
PinuiD : FDNFVEGLYGFAKRHSYLGKDGMDN-TRYSDLLKNAISL- : 123
PknoC  : FDNIVQGFYGFAKRHNYISQEQMNN-TSHTNLLKNAINL- : 115
PcoaC  : FDNFVQGLYGFAKRHNYLGKERMDNTTSYSNLLKNALSL- : 124
PberB  : FKNFIYGVYSFAKKYNYLSESRLEE--EN----------- : 109
PyoeB  : FKNFIYGIYSFAKKYNYLSATRLAE--ANSQFIPNVLK-- : 118
PchaB  : FKNFIYGIYSFAKKHNYLSTARLAE--ENSQFISNVLNVL : 119
PvinvB : FKNFIYVIYSFAKKYNYLSKTRLED--ENKQFITNVLN-- : 118
PvinpB : FKNFIYGIYSFAKKYNYLSKTRLAE--ENSQFIRNVLN-- : 118
         F Nf6 g Y FAK4 nY6   r6             n
```

Comparison 28: Clade 4 Rodent-parasites vs Clade 2 Rodent-parasites

```
                  *        20         *        40         *   +    60         *        80
PberB  : -AFLGTIFDEILNEQNHNEQVHTTQYHSKYNSLKSECD--FAMNLEEYAIAKK-ISSYFK--SGTAENPIYLYDILIKSLNDEEYK :  80
PyoeB  : -AFLGAIFDEILKEQHHDDDVHSTEYHSKYNSLKNECD--FPMNLEEYNIAKK-ISSYFQ--SGSTENPIYLYDILLKSLKDEEYK :  80
PchaB  : -AFLGTVFDEMLKAQDHAKKVHTNEYHSKYNALKGECD--FAMSLEEYEIAKK-ISTYF---NGTTENPIHLYDILIKAITDEEYK :  79
PvinvB : -AFLGTIFDDMLKEQNGNTTINNSEYHSKYNDLKTECD--LSMSTDEYDIAKKLISTYF---SGTTEHPIKLYDILVKAITDEEYK :  80
PvinpB : -AFLGTVFDEMLKEQKHDTKIHNTEYHSKYNVLKTECD--FAMSIEEYDIAKKLISTYF---RGTTDNPIHLYDILIKAITDEEYK :  80
PberC  : -NYTNDFYNAVIASLNEND--KNGNYKNKYEDFKNKYDNVIAINEKEFEIFSK-VEVFMKNHHDFNLNSNFLYELLTKALSDQNFK :  82
PyoeC  : -NYTNDFYNAVIANLNEND--KNGNYKNKYEDFKNKYDNVIAIDEKEFEIFSKLVEVFMKNHQDFSFNSNFLYELLTKALSDQGFK :  83
PchaC  : QNYTDDFFNSVIANINEND---NGDYKDKYEDFKSKYDNIIAINQKEFEIFSK-IEGFMQSNKDMNLSSDYLYEVLTKALSDKAFK :  82
PvinvC : -NYADDIFGSVIANINTSD---NGDYKVKYEDFKNKYDNLIAIDQTEFEIFSKLVEGFMKTNKEINLSSDYLYDVLTKTLSDKAFK :  82
PvinpC : -NYTDDFFNSVIANINTSD---NGEYKIKYEDFKNKYDNIMAIDQREFEIFSKLVEGFMKNNKEMNLSSDYLYEVITKALSDTAFK :  82
           5    5  66             Y  KY   K   D   a6   E5 I  K 6  5             LY 66 K 6 D  5K

            *+      100         *       120
PberB  : KHFKNFIYGVYSFAKKYNYLSESRLEEEN------------- : 109
PyoeB  : KHFKNFIYGIYSFAKKYNYLSATRLAEANS--QFIPNVLK-- : 118
PchaB  : KHFKNFIYGIYSFAKKHNYLSTARLAEENS--QFISNVLNVL : 119
PvinvB : KHFKNFIYVIYSFAKKYNYLSKTRLEDENK--QFITNVLN-- : 118
PvinpB : KHFKNFIYGIYSFAKKYNYLSKTRLAEENS--QFIRNVLN-- : 118
PberC  : TEFKEFMNNMYNFVKKNQE-GKVMTDNDKMYMALFENVLSL- : 122
PyoeC  : TEFKEFMNNMYNFVKKKQE-GKPMTENDKIYMTLFENVLSL- : 123
PchaC  : AEFKEFMNSMYNFIKKKHE-GKTLSENDKQYMALFENVLTL- : 122
PvinvC : DEFKSFMNNMYNFVKKKHE-GKLTTETDKQYMALFENVLSL- : 122
PvinpC : AEFKDFMNNMYSFVKKKHE-GKTLSESDKQYMTLFENVLSL- : 122
           FK F6  6Y F KK                    nvl
```

In pairwise comparisons with statistical significant θ_D values, DIVERGE computes a posterior probability for detecting putative amino acids responsible for such divergence. Those putative sites were highlighted in green. In addition, the sites at C-terminal region (MSP_7C domain) under positive selection found by MEME method were tagged with a red plus symbol (+). Since, functional divergence could involve relaxation of functional constraint or could be due positive selection a perfect correlation between putative amino acids responsible for divergence and positive selection is not expected. Positive selection might be involved in the acquisition of a new role but also could be the outcome of adaptation to new host during *Plasmodium* evolutionary history. Positive selected sites were inferred using the clade (e.g sequences within clade 1) and not for the comparison (e.g Clade 1 Primate-parasites vs Clade 2 Primate-parasites).
